# Butterfly Diversity in a Sacred Kaya Forest in Southern Kenya

**DOI:** 10.1002/ece3.73242

**Published:** 2026-03-20

**Authors:** Laura Wagner, Jan Christian Habel, Maria Fungomeli, Mike Teucher, Thomas Schmitt

**Affiliations:** ^1^ Senckenberg – Leibniz Institution for Biodiversity and Earth System Research Senckenberg German Entomological Institute Müncheberg Germany; ^2^ University of Potsdam Institute of Biochemistry and Biology Potsdam Germany; ^3^ Evolutionary Zoology, Department of Environment and Biodiversity University of Salzburg Salzburg Austria; ^4^ Coastal Forests Conservation Unit Centre for Biodiversity, National Museums of Kenya Kilifi Kenya; ^5^ Department of Geoecology, Institute for Geosciences and Geography Martin Luther University Halle‐Wittenberg Halle/Saale Germany

**Keywords:** biodiversity hotspot, butterflies, generalist species, habitat mosaic, seasonality, specialist species, transect counts

## Abstract

Habitat destruction and the deterioration of habitat quality are among the main drivers causing worldwide biodiversity loss. The lowland tropical dry forests of the East African coastal region, a hotspot of endemism, have been negatively affected by anthropogenic activities over the last few centuries. Today, only small remnants of these forests with their pristine flora and fauna still exist. It is questionable to what extent the original biodiversity can persist in such small and isolated habitat remnants. Butterflies respond highly sensitively to environmental changes. In this study, we analysed butterfly community structures across a habitat mosaic consisting of natural ecosystems and anthropogenic agro‐environments in southern Kenya. Butterflies were counted along line‐transects in dense forest, along the forest margin, and in neighbouring pastures and orchards; several biotic and abiotic parameters were assessed for all transect sections (e.g., addressing the vegetation structure). Data collection was conducted during the rainy and dry season, as well as during the transition between both seasons. We compiled species specific traits on the butterfly species ecology, distribution, and behaviour. The obtained results revealed significant differentiation among the butterfly communities in natural forest, forest margins, and in the anthropogenic ecosystems. Although both butterfly diversity and abundance were lowest inside the natural forest, vulnerable forest specialist species occurred restricted to the forest and were absent from anthropogenic ecosystems. The butterfly communities found in the agro‐environments were species‐rich if compared with the natural forest ecosystem, but dominated by generalist species. From the dry to the rainy season, species numbers increased in all habitat types, but the number of individuals increased only at forest margins and in pastures. This underlines the buffering effect against drought in forests but also in orchards. In general, our data underline that no surrogate habitat exists for typical forest butterflies. This underlines the high ecological relevance of such forest remnants and confirms the necessity of strict conservation of these last remnants of lowland dry forest.

## Graphical abstract

In this study we analysed the butterfly communities in a tropical coastal forest patch and the surrounding anthropogenic ecosystems. Although both abundance and species richness were lowest inside the natural forest, vulnerable forest specialists were restricted to the forest interior and absent from all anthropogenic landscapes. The butterfly communities found in the agricultural landscapes were comparatively species rich, and single generalist species were found in high numbers. Neither in the orchards nor in pastures could typical forest butterflies be detected. Thus, there exist no surrogate habitats for forest butterflies. This underlines the relevance of these forest remnants to preserve biodiversity in the long run.

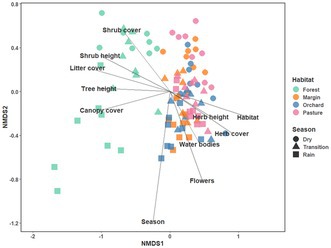

## 1 Introduction

The destruction of natural habitats and the deterioration of habitat quality are among the main threats to biodiversity across the globe (Foley et al. 2005). Demographic pressure and increasing life standards cause rising demand for resources and land, which causes the destruction and fragmentation of pristine ecosystems (Maxwell et al. 2016; Leisher et al. 2022). These human interventions have led to the extinction of many species, the vanishing of local populations, and changes in species community composition (Lindenmayer and Fischer 2006). Thus, small and geographically isolated habitat remnants harbour only a fraction of the original species diversity (Gascon et al. 1999; Barlow et al. 2007; Nogueira et al. 2021). At the same time, it has also been demonstrated that small and geographically isolated habitat patches may still provide space and resources for many species and thus may hold (at least a large proportion of) the original biodiversity. And, even anthropogenic habitats and highly modified ecosystems such as secondary forests and various agro‐environments may act as surrogate habitats for a large number of moderately specialized species (Chazdon 2014).

In the tropics, natural habitats in particular harbour a large number of species, including many specialist species in high abundances compared with disturbed and/or human‐made ecosystems (Brockerhoff et al. 2008), as also shown for butterflies (Schulze et al. 2010a, 2010b; Habel et al. 2018), and birds (Waltert et al. 2004). However, the total species richness and abundance of species in human‐made ecosystems might also be high if compared with natural ecosystems, exemplified for butterflies (Kudavindanage et al. 2012), bees (Winfree et al. 2009), and birds (Mulwa et al. 2012, 2021). This is mostly due to high habitat heterogeneity, as found in gardens or extensively used agro‐environments, such as pastures or orchards.

In this context, habitat demand and the degree of ecological specialisation of the respective species has to be considered. Habitat specialist species with restricted movement are usually found exclusively in largely intact natural ecosystems and are quickly disappearing from ecosystems when they get disturbed by human activities (Koh and Sodhi 2004). On the other hand, the availability of specific resources (such as blossoms, fruits, and water) may attract many organisms, even to human‐made ecosystems and disturbed habitats (Kudavindanage et al. 2012; Calviño‐Cancela 2013; Schmitt et al. 2020). However, ecologically demanding species mostly cannot successfully develop in disturbed anthropogenic habitats. Consequently, these ecosystems at maximum represent surrogate habitats for adult individuals of specialists, and for more generalist species (Dennis et al. 2006; Habel et al. 2018; Schmitt et al. 2020). In addition, seasonal effects also impact abundance and community composition (Habel et al. 2018).

East Africa is known for its vast diversity of flora and fauna, including endemics, which can be found mostly in forest ecosystems, such as in the East African coastal forests (Burgess and Clarke 2000; Myers et al. 2000). Because of demographic pressure and commercial forestry, most of the pristine forest has been destroyed since the colonial era, and only a few, mostly small and geographically isolated forest remnants, exist today (Burgess and Clarke 2000; Fungomeli et al. 2020; Ngumbau et al. 2020). Continuing forest destruction negatively impacts the unique biodiversity still existing in forest remnants (Burgess et al. 2003; Choudhary and Chishty 2020). Meanwhile, most of the remaining forest patches today are under strict ecosystem conservation or are preserved as cultural heritage sites, such as the sacred Kaya forests in southern Kenya (Matiku 2005). However, the habitat quality of these remaining forest patches continues to degrade because of illegal resource exploitation, such as selective logging, charcoal production, collection of deadwood, and poaching (Matiku 2005). As a result, many of the remaining forest habitats in East Africa are of low habitat quality (Kibet and Nyamweru 2008; Osewe et al. 2022; Waltert et al. 2005, 2011).

Butterflies represent excellent model organisms, as many of them respond highly sensitively to environmental changes, such as habitat modifications and seasonality (Fleishman and Murphy 2009; Habel et al. 2018; Legal et al. 2020; Schmitt et al. 2021). Therefore, we analysed butterfly diversity and species community composition across a habitat mosaic consisting of natural forest and anthropogenic ecosystems such as forest margins, extensively used pastures, arable fields, and orchards. We counted butterflies along line‐transects during the dry and rainy season and assessed specific habitat parameters for each transect section. In addition, we compiled trait data on the ecology and distribution for each butterfly species observed in the field. On the basis of our obtained results, we address the following questions:

1. Do butterfly diversity, butterfly abundance, and community structure differ between natural forest and the adjoining anthropogenic ecosystems, and how are these impacted by the respective habitat parameters?
2. Do butterfly diversity, butterfly abundance, and community structures vary among seasons and how strong is their impact in the different habitat types?
3. Do species traits reflect the different habitat types and how strongly do they differ?
4. Do modified habitats act as alternative or surrogates for specialised forest species?

## 2 Material and Methods

### 2.1 Study Area

**FIGURE 1:**
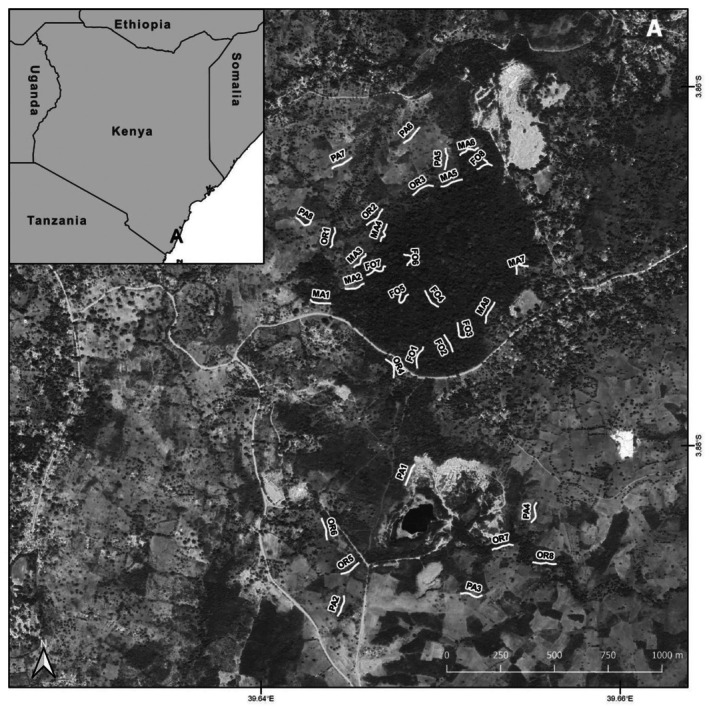
Study area in southern Kenya (A in the small inlet map), and the location of each transect line in the study area (large map). Transect numbers correspond with all figures and tables. The map was created using the programs QGIS and GIMP. Abbreviations of habitat types: FO—forest; MA—forest margin; OR—orchard; PA—pasture.

Our study area covers a habitat mosaic of natural forest and human‐made ecosystems in south‐eastern coastal Kenya (Kilifi County) (Figure 1). The studied natural forest patch represents 0.75 km^2^ of lowland tropical dry coastal forest, but strongly suffers from severe resource exploitation as illegal logging, wood‐harvesting, charcoal production, and hunting (Kibet 2011). It is a sacred Kaya forest and as such one of the 107 remaining lowland dry forest fragments, which still exist along the Kenyan coast (Burgess and Clarke 2000). The nearest fragments of natural dry forest are in close geographic proximity (Kaya Ribe to the south‐west about 3.9 km distant, and Kaya Jibana 3.8 km to the north‐east). The forest patch is surrounded by orchards (with mango, cashew, and coconut trees), pastures, and settlement areas, but also few small plantations (mainly eucalyptus). All these types of ecosystems form a habitat mosaic (Figure 1).

The climate of this region is hot year‐round (with mean temperatures of about 25.5°C) (Jaetzold et al. 2010) but is characterised by strong seasonal shifts. The two rainy seasons last approximately from end‐March to July (average seasonal rainfall about 650 mm) and from October to December (average rainfall about 300 mm) (Jaetzold et al. 2010).

### 2.2 Transect Counts

We established 32 line transects (with 100 m each) in the following four habitat types: Natural forest, forest margin, pasture, and orchard. Eight transects were placed in each of these four habitat types (Figure 1). Minimum distance among single‐line transects was 100 m to reduce potential effects from autocorrelation. However, especially large and mobile butterfly species might be sufficiently dispersive to exchange among transects. However, it can be assumed that the effect is rapidly decreasing with distance.

Butterfly observations were performed from 1 March to 16 May 2022. During 60 days of field work, each transect was visited 20 times in total, with mostly 3–5 days between visits (min: 2; max: 8 days). Transect walks were conducted in the time from 08:30 am to 04:00 pm under sunny or partly cloudy weather conditions with temperatures > 20°C. As butterfly activity strongly relies on daytime, all transects were walked rotationally at different day times. The only exception from this rule was transects pasture 5, 6, and 7. These Transects are all located on a hilly top with strong sun exposure. Thus, these transects were always visited during the morning hours to avoid the afternoon heat, which strongly reduces the activity of most butterflies and thus would strongly bias our sampling.

Transect walks were conducted at a slow pace, and all butterflies observed within a 5 m buffer at each side (left, right, in front, above) of the transect line were determined and noted (species and individuals) (Pollard 1977; Pollard and Yates 1993, modified). Whenever possible, butterflies were determined to the species level in the field. If necessary, individuals were netted, photographed, and determined afterwards (on the basis of Larsen 1991, 2005). At the beginning of each transect walk, temperature, percentage of cloud cover, wind speed, and total precipitation (in mm, on the basis of a home‐made rain gauge) were measured or estimated and noted (Appendix S4). Since the first heavy rains arrived after eight sampling rounds in the afternoon of 29 March, this date represents the end of the dry season. The subsequent four transect visits (1–13 April) represent the transition from dry to rainy season, and the last eight transect walks (14 April–16 May) cover the rainy season. Raw data are given in Appendix S1.

### 2.3 Habitat Parameters

Habitat parameters that may affect the occurrence of butterfly species and their abundance were assessed at the start, middle, and end points of each transect (0 m, 50 m, and 100 m). We recorded the following parameters: Percentage of canopy cover (estimated visually and classified into percentage shares in steps of 5%), height of the tallest tree (visual estimate using parallax distance measurement and subsequently classified into metres), percentage of shrub cover, mean shrub height (m), percentage of herb cover, mean herb height (cm), percentage of litter cover, and number of blossoms of herbs. Estimates were done for a 10 m radius of the respective starting point, midpoint, and ending point (see above). Additionally, it was noted whether a creek (also if dry) was present within a distance of 20 m from the transect line. The occurrence of all types of blossoms producing nectar was classified into five classes of occurrence: 0 = no flower, 1 = 1–9 flowers, 2 = 10–48, 3 = 50–99, 4 = ≥ 100. For composite inflorescences, we considered each single blossom. To account for changes between seasons, the number of blossoms was reassessed during each transect walk and a mean value was calculated for each transect. All other parameters were assessed only once per season (i.e., three times in total). Given the nature that some of the habitat traits are corrected, this was taken into account during the statistical analysis. All data of habitat parameters are given in Appendix S1, their means per habitat type are compiled in Appendix S4.

### 2.4 Butterfly Traits

All butterfly species observed were classified depending on their ecology and distribution. In total, we used eight traits: Distribution (five classes from “Kenyan endemic” to “distributed across the African continent and beyond”); Phagy of larvae (three classes, from monophagous to polyphagous); Larval food‐plant type (three classes, mainly grasses and herbs—both—mainly bushes and trees); Larvae feeding on lichens/algae (no/yes); Hemeroby (four classes, from “only natural habitats” to “more common in anthropogenic than in natural habitats”); Water dependency (three classes: occurring in truly arid habitats—accepting intermediate arid habitats—not occurring in arid habitats); Savannah index (five classes, from “not occurring in savannahs” to “only in savannahs”); Tree dependency (three classes: not requiring trees—requiring trees—exclusively in forests). Details about the eight traits are given in Appendix S2. Classifications were adopted from literature, such as Larsen (1991, 2005), Schmitt et al. (2020, 2021), and from online databases (metamorphosis.org.za, learnaboutbutterflies.com). Similar to habitat parameters, correlations between some butterfly traits were expected and investigated statistically. Classifications for single species are compiled in Appendix S1.

### 2.5 Statistical Analyses

Sampling results were arranged into a 96‐transect x 94‐species table, representing each transect for the observed season (dry, transition, rainy). Abundances were summed up for each single species and per season and transect. Additionally, mean abundances and species richness were calculated. The Online software “iNext Online” (Chao et al. 2014, 2016) was used to test the sample completeness of each habitat and season. All other statistical analyses were performed with R (version 4.2.1, R Core Team 2022). Using R, Shannon indices as well as inverted Simpson Indices and Species Evenness were calculated to quantify butterfly diversity for each habitat and season. Kruskal–Wallis *H* tests were then performed to compare species richness, abundance, diversity, and species traits as well as habitat parameters among seasons and habitats, as well as within seasons and habitats. If results were significant, pairwise Wilcoxon rank sum tests were used to obtain pairwise comparisons. Linear models were performed to assess the influence of habitat and season on butterfly diversity and traits. In order to avoid multicollinearity, Pearson correlation coefficients were computed for the dependent variables. If multiple variables were correlated by more than *r* = 0.7, all but one of them were removed. To prevent a disproportional influence of highly abundant species on the models, community‐weighted mean species trait values were calculated on the basis of ln‐transformed abundance data. NMDS was performed on ln‐transformed abundances using Bray‐Curtis dissimilarities and, to provide an alternate perspective on the data and potentially help validate observed clusters, NMDS was followed by a cluster analysis. A Mantel test using Spearman's correlations was performed to test the correlation between our faunal matrix and our topographic distance matrix. More details on statistical analyses are given in Appendix S3.

## 3 Results

### 3.1 Abundance and Diversity

**FIGURE 2:**
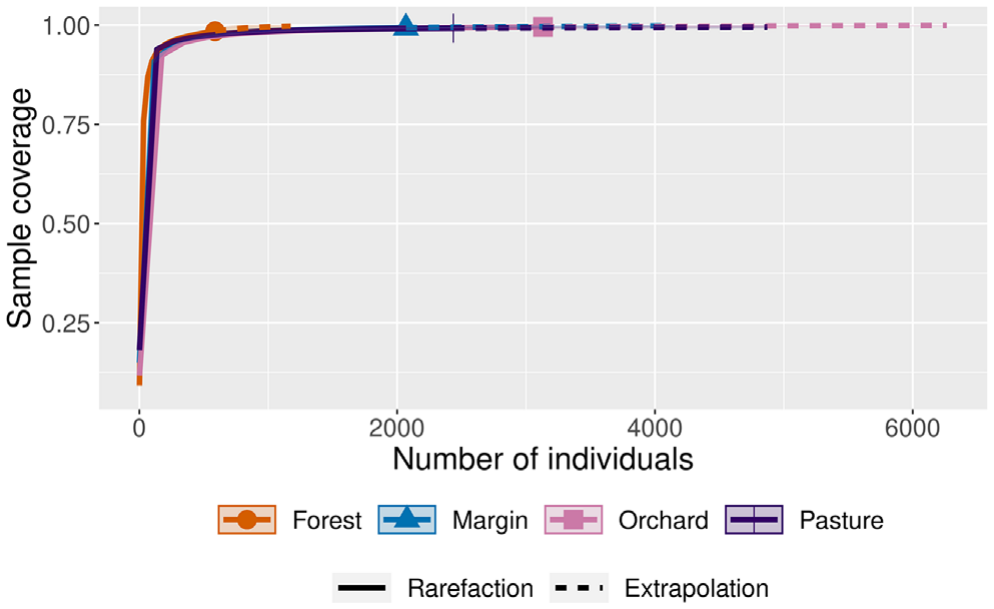
Sample completeness curve estimating the sample coverage of the four studied habitats with a confidence interval of 95%. Number of bootstraps = 1000, q = 1.

In total, 8223 individuals of 94 butterfly species were recorded; raw data are given in Appendix S1. With 76 species and 3132 individuals, orchards contained the highest total species richness and abundance. Pastures were second in abundance (2436), followed by forest margins (2068). By contrast, species richness was second highest at forest margins (62), followed by pastures (58). Only 42 species and 587 individuals were observed in the forest, making this habitat the poorest in richness and abundance (Table 1). Our sample completeness curve showed sample coverage to be close to complete and to differ little between habitat types (Figure 2). Results of non‐parametric Kruskal–Wallis ANOVAs showed that differences among habitat types were significant for abundance (H(3) = 45.36, *p* < 0.001), mean species richness (H(3) = 40.45, *p* < 0.001), Shannon index (H(3) = 11.31, *p* = 0.01), and Evenness (H(3) = 28.89, *p* < 0.001), but not Simpson index (H(3) = 6.41, *p* = 0.09) (Table 1, Appendix S4).

**TABLE 1:** Diversity and abundance of butterfly communities across habitats and seasons.

Variable	All habitats	Forest	Forest margin	Orchard	Pasture
All seasons					
S_Tot_	94	42	62	76	58
S_S_	5.06 (± 0.27)	2.48 (± 0.25)^a^	5.41 (± 0.40)^b^	7.18 (± 0.52)^c^	5.18 (± 0.44)^b^
A_Tot_	8223	587	2068	3132	2436
A_Tr_	106.1 (± 9.0)	30.6 (± 3.8)^a^	109.5 (± 14.5)^b^	158.4 (± 21.2)^b^	126.0 (± 16.4)^b^
H_Tr_	1.91 (± 0.04)	1.8 (± 0.09)^ab^	1.98 (± 0.07)^ab^	2.09 (± 0.08)^b^	1.77 (± 0.07)^a^
D_Tr_	0.77 (± 0.01)	0.77 (± 0.02)^a^	0.79 (± 0.01)^b^	0.79 (± 0.02)^b^	0.74 (± 0.02)^b^
E_Tr_	0.77 (± 0.01)	0.86 (± 0.01)^a^	0.76 (± 0.02)^a^	0.75 (± 0.02)^a^	0.72 (± 0.02)^a^
Dry season					
S_Tot_	64	24	29	47	27
S_S_	3.86 (± 0.40)^A^	2.41 (± 0.38)^aA^	3.75 (± 0.52)^abA^	5.98 (± 1.1)^bA^	3.28 (± 0.48)^abA^
A_Tot_	2621	229	530	1213	649
A_Tr_	81.9 (± 15.7)^A^	28.6 (± 5.6)^aA^	66.3 (± 11.0)^abA^	151.6 (± 48.6)^bA^	85.1 (± 26.6)^abA^
H_Tr_	1.77 (± 0.06)^A^	1.77 (± 0.12)^aA^	1.81 (± 0.11)^aA^	1.89 (± 0.12)^aA^	1.61 (± 0.15)^aA^
D_Tr_	0.75 (± 0.01)^A^	0.76 (± 0.03)^aA^	0.77 (± 0.02)^aA^	0.74 (± 0.03)^aA^	0.71 (± 0.03)^aA^
E_Tr_	0.75 (± 0.02)^A^	0.82 (± 0.03)^aA^	0.77 (± 0.03)^abA^	0.69 (± 0.02)^bA^	0.73 (± 0.04)^abA^
Transition					
S_Tot_	55	22	30	38	32
S_S_	5.50 (± 0.46)^B^	2.69 (± 0.43)^aA^	5.78 (± 0.68)^bB^	7.53 (± 0.87)^bA^	6 (± 0.76)^bB^
A_Tot_	1965	147	559	670	589
A_Tr_ *	122.8 (± 16.9)^B^	36.8 (± 6.7)^aA^	139.8 (± 34.6)^bA^	167.5 (± 39.5)^bA^	147.3 (± 28.1)^bA^
H_Tr_	1.83 (± 0.07)^A^	1.62 (± 0.16)^aA^	1.87 (± 0.8)^aA^	2.07 (± 0.13)^aA^	1.77 (± 0.11)^aA^
D_Tr_	0.77 (± 0.02)^AB^	0.75 (± 0.03)^aA^	0.78 (± 0.03)^aA^	0.80 (± 0.05)^aAB^	0.74 (± 0.03)^aA^
E_Tr_	0.8 (± 0.02)^A^	0.87 (± 0.02)^aA^	0.78 (± 0.03)^abA^	0.80 (± 0.05)^abA^	0.74 (± 0.03)^bA^
Rainy season					
S_Tot_	78	33	50	57	46
S_S_	5.83 (± 0.46)^B^	2.36 (± 0.55)^aA^	6.69 (± 0.47)^bA^	8.02 (± 0.63)^bA^	6.25 (± 0.6)^bB^
A_Tot_	3637	211	979	1249	1198
A_Tr_	113.7 (± 13.4)^B^	26.4 (± 7.8)^aA^	122.4 (± 18.5)^bA^	156.1 (± 22.6)^bA^	149.8 (± 27.1)^bA^
H_Tr_	2.11 (± 0.07)^B^	1.92 (± 0.17)^aA^	2.27 (± 0.08)^aB^	2.32 (± 0.11)^aA^	1.94 (± 0.11)^aA^
D_Tr_	0.81 (± 0.01)^B^	0.80 (± 0.03)^aA^	0.83 (± 0.02)^aA^	0.84 (± 0.02)^aB^	0.76 (± 0.04)^aA^
E_Tr_	0.77 (± 0.02)^A^	0.88 (± 0.02)^aA^	0.74 (± 0.03)^bA^	0.76 (± 0.02)^bA^	1.68 (± 0.02)^bA^

*Note:* Shown are the total species richness (S_Tot_) and abundance (A_Tot_) as well as the mean species richness per sampling round and transect (S_S_), abundance per transect (A_Tr_), Shannon index per transect (H_Tr_), Simpson index per transect (D_Tr_), and Evenness per transect (E_Tr_). Errors refer to the conventional standard error of the mean. Different letters denote statistically significant differences, with lowercase letters describing differences among habitats (rows) and capital letters describing differences among seasons (columns).

* The abundance per transect was multiplied with two for the transition period to compensate for the 50% lower number of walks during this period.

Abundance and species richness increased from dry (328 individuals as mean per sampling round; 64 species total) to rainy season (455; 78); 491 individuals as mean per round, with a total of 55 species were recorded during the transition period. Mean species richness (H(2) = 13.3, *p* = 0.001), abundance (H(2) = 7.07, *p* = 0.03), as well as Shannon (H(2) = 14.33, *p* < 0.001) and Simpson index (H(2) = 8.94, *p* = 0.01) differed significantly among seasons, but not Evenness (H(2) = 4.71, *p* = 0.09). Numerous significant pairwise differences were detected within the significant parameters (Table 1).

Seasonal effects were similar within forests and orchards, that is, total species numbers increased towards the rainy season, but abundance remained mostly unchanged. Respective means did not differ significantly among seasons (all *p* ≥ 0.3). Likewise, forest margins and pastures had similar patterns, i.e., total species numbers were also highest during the rainy season, but abundance also increased strongly from the dry season to the transition and rainy season. Significant differences were obtained in the minority of cases for the other diversity parameters assessed (Table 1).

Linear models revealed a significant correlation between species richness and habitat characteristics (*p* < 0.001, *R* ^2^ = 0.53). Species richness was significantly and positively affected by the presence of water bodies (ß = 0.23, *p* = 0.03, *R* ^2^ = 0.05), the number of flowers (ß = 0.45, *p* < 0.001, *R* ^2^ = 0.23), and season (ß = 0.17, *p* = 0.02, *R* ^2^ = 0.06) (Appendix S4). Similarly, butterfly abundance was correlated to habitat traits (*p* < 0.001, *R* ^2^ = 0.53), with the number of flowers significantly affecting abundance (ß = 0.45, *p* < 0.001, *R* ^2^ = 0.27). There was a low correlation between the Simpson index and habitat traits (*p* = 0.14, *R* ^2^ = 0.04), with the Simpson Index being significantly affected by season (ß = 0.23, *p* = 0.03, *R* ^2^ = 0.05) (Table 2). Details see Appendix S5.

**TABLE 2:** Mean species traits of the butterfly communities across habitat types and seasons.

Trait	All habitats	Forest	Forest margin	Orchard	Pasture
All seasons					
Distribution	4.29 (± 0.04)	3.90 (± 0.09)^a^	4.34 (± 0.05)^b^	4.35 (± 0.04)^b^	4.57 (± 0.03)^c^
Larval diet breadth	2.60 (± 0.01)	2.78 (± 0.03)^a^	2.54 (± 0.02)^b^	2.54 (± 0.01)^b^	2.53 (± 0.02)^b^
Larval food plant type	2.33 (± 0.03)	2.64 (± 0.04)^a^	2.26 (± 0.03)^b^	2.18 (± 0.04)^b^	2.22 (± 0.04)^b^
Lichens/algae	1.05 (± 0.01)	1.16 (± 0.03)^a^	1.03 (± 0.01)^b^	1.01 (± 0.00)^b^	1.00 (± 0.00)^c^
Hemeroby index	3.14 (± 0.04)	2.71 (± 0.12)^a^	3.23 (± 0.06)^b^	3.17 (± 0.04)^b^	3.43 (± 0.04)^c^
Savannah index	2.89 (± 0.06)	2.03 (± 0.09)^a^	3.05 (± 0.06)^b^	3.00 (± 0.07)^b^	3.47 (± 0.03)^c^
Water index	1.66 (± 0.03)	2.02 (± 0.06)^a^	1.58 (± 0.04)^b^	1.64 (± 0.03)^b^	1.39 (± 0.03)^c^
Tree index	1.54 (± 0.04)	2.00 (± 0.09)^a^	1.46 (± 0.05)^b^	1.51 (± 0.05)^b^	1.18 (± 0.02)^c^
Dry season					
Distribution	4.24 (± 0.07)^A^	3.74 (± 0.16)^aA^	4.32 (± 0.10)^abA^	4.37 (± 0.06)^bA^	4.51 (± 0.04)^bA^
Larval diet breadth	2.55 (± 0.03)^A^	2.74 (± 0.05)^aA^	2.48 (± 0.03)^bA^	2.51 (± 0.03)^bA^	2.46 (± 0.04)^bA^
Larval food plant type	2.26 (± 0.05)^A^	2.64 (± 0.09)^aA^	2.12 (± 0.05)^bA^	2.12 (± 0.05)^bA^	2.19 (± 0.09)^bA^
Lichens/algae	1.07 (± 0.02)^A^	1.22 (± 0.04)^aA^	1.05 (± 0.03)^bA^	1.01 (± 0.01)^bA^	1.00 (± 0.00)^bA^
Hemeroby index	3.19 (± 0.06)^A^	2.79 (± 0.13)^aA^	3.29 (± 0.13)^abAB^	3.21 (± 0.04)^abA^	3.45 (± 0.06)^bA^
Savannah index	3.04 (± 0.10)^A^	2.20 (± 0.11)^aA^	3.27 (± 0.09)^bA^	3.14 (± 0.11)^bA^	3.54 (± 0.04)^cA^
Water index	1.60 (± 0.05)^A^	1.96 (± 0.07)^aA^	1.49 (± 0.09)^bA^	1.59 (± 0.06)^bA^	1.36 (± 0.05)^bA^
Tree index	1.45 (± 0.07)^A^	1.92 (± 0.11)^aA^	1.30 (± 0.07)^bA^	1.42 (± 0.10)^bcA^	1.14 (± 0.04)^cA^
Transition					
Distribution	4.43 (± 0.05) ^A^	4.23 (± 0.14) ^aA^	4.48 (± 0.06) ^aA^	4.38 (± 0.05) ^aA^	4.61 (±0.08) ^aA^
Larval diet breadth	2.65 (± 0.03) ^A^	2.87 (± 0.04) ^aA^	2.61 (± 0.02) ^bB^	2.56 (± 0.03) ^bA^	2.55 (± 0.02) ^bA^
Larval food plant type	2.33 (± 0.04) ^A^	2.63 (± 0.03) ^aA^	2.33 (± 0.03) ^bB^	2.18 (± 0.05) ^bA^	2.19 (± 0.06) ^bA^
Lichens/algae	1.04 (± 0.01) ^A^	1.11 (± 0.05) ^aA^	1.03 (± 0.01) ^aA^	1.00 (± 0.003) ^aA^	1.00 (± 0.00) ^aA^
Hemeroby index	3.34 (± 0.05) ^A^	3.22 (± 0.14) ^aB^	3.41 (± 0.08) ^aA^	3.26 (± 0.05) ^aA^	3.48 (± 0.07) ^aA^
Savannah index	2.95 (± 0.08) ^A^	2.29 (± 0.09) ^aA^	3.04 (± 0.07) ^bAB^	3.04 (± 0.09) ^bA^	3.44 (± 0.06) ^cA^
Water index	1.59 (± 0.05) ^A^	1.87 (± 0.09) ^aA^	1.55 (± 0.06) ^bcA^	1.61 (± 0.04) ^bA^	1.34 (± 0.05) ^cA^
Tree index	1.43 (± 0.05) ^A^	1.69 (± 0.11) ^aA^	1.38 (± 0.07) ^bcA^	1.50 (± 0.08) ^bA^	1.14 (± 0.04) ^cA^
Rainy season					
Distribution	4.21 (± 0.07) ^A^	3.73 (± 0.13) ^aA^	4.24 (± 0.04) ^bA^	4.30 (± 0.08) ^bA^	4.60 (± 0.05) ^cA^
Larval diet breadth	2.60 (± 0.02) ^A^	2.72 (± 0.05) ^aA^	2.54 (± 0.03) ^bAB^	2.57 (± 0.02) ^bA^	2.59 (± 0.03) ^abA^
Larval food plant type	2.38 (± 0.04) ^A^	2.67 (± 0.06) ^aA^	2.32 (± 0.04) ^bAB^	2.26 (± 0.07) ^bA^	2.28 (± 0.08) ^bA^
Lichens/algae	1.05 (± 0.02) ^A^	1.14 (± 0.06) ^aA^	1.02 (± 0.01) ^aA^	1.02 (± 0.01) ^aA^	1.00 (± 0.00) ^aA^
Hemeroby index	2.89 (± 0.09) ^B^	2.12 (± 0.12) ^aC^	3.00 (± 0.05) ^bB^	3.05 (± 0.10) ^bcA^	3.36 (± 0.07) ^cA^
Savannah index	2.67 (± 0.13) ^A^	1.59 (± 0.11) ^aB^	2.84 (± 0.07) ^bB^	2.82 (± 0.13) ^bA^	3.45 (± 0.04) ^cA^
Water index	1.78 (± 0.06) ^A^	2.23 (± 0.09) ^aA^	1.70 (± 0.03) ^bA^	1.72 (± 0.06) ^bA^	1.46 (± 0.03) ^cA^
Tree index	1.75 (± 0.08) ^B^	2.41 (± 0.11) ^aB^	1.69 (± 0.09) ^bB^	1.63 (± 0.09) ^bA^	1.27 (± 0.04) ^cA^

*Note:* Errors are conventional standard errors of the mean. Different letters denote statistically significant differences with lowercase letters describing differences among habitats (rows) and capital letters describing differences among seasons (columns).

These seasonal fluctuations were also reflected in strongly differing phenologies of the individual species with four major groups distinguished. Thus, (i) several species (e.g., *Junonia hierta*, *Tagiades flesus*, *Graphium antheus*, and *Melanitis leda*) were absent or rare during the dry season, but showed a clear increase in abundance during or shortly after the transition Appendix S4. Opposed to this, (ii) the abundances of other species (e.g., *Byblia ilithyia*, *Colotis evagore*, *Eurema hecabe*, and *Balichila minima*) decreased over the course of the study and reached their lowest numbers during the rainy season (Appendix S4). (iii) A third group of species (e.g., *Junonia oenone*, *Belenois thysa*, *Eurytela dryope*, and *Baliochila hildegarda*) displayed an initial strong decrease in abundance from the first sampling round towards the first rains, followed by a rapid increase; abundances continued increasing until the beginning of the rainy season when they again began to moderately decrease (Appendix S4). (iv) Further species (e.g., *Danaus chrysippus, Hypolimnas misippus, Andronymus neander*, and *Bicyclus safitza*) were similar but reached their peak earlier (i.e., in the middle of the transition period) (Appendix S4). Dominance patterns largely followed the development of species' individual abundances.

### 3.2 Community Structure and Trait Differences Among Habitats and Seasons

Most of the 94 recorded species were widespread beyond East Africa; only one species was a Kenyan endemic (i.e., *Baliochila minima*), and eight were endemic to East Africa. The communities differed significantly among habitat types in the geographic distribution of their species (H(3) = 38.55, *p* < 0.001), with the smallest mean distributions in forests and the widest on pastures. Significant differences (all *p* < 0.001) among the habitats' communities were also obtained for all other traits assessed. Larval diet breadth, the proportion of species feeding on lichen and/or algae, the proportion of species feeding on woody plants, water dependency, and tree index were highest in the forest and lowest on pastures; the reverse was observed for the savannah index. Forest margins and orchards always had intermediate positions and never differed significantly from each other.

Trait composition did not change remarkably over seasons, and significant changes (*p* < 0.05) were only obtained for the rainy season with a decrease in the hemeroby index and increase in the tree index. The pattern found for the entire data set was also obtained within the three seasons (Table 2). Several habitat and species traits were found to be highly correlated, that is, canopy cover and litter cover (*r* = 0.81), water index and savannah index (r = −0.9) as well as hemeroby and distribution (*r* = 0.87).

Linear models detected larval diet breadth (i.e., their community weighted means; species feeding on lichens and mosses were excluded as their diet breadth is unknown) to be significantly affected by habitat traits (*p* < 0.001, *R* ^2^ = 0.37 ± 0.006), with a significant positive effect of tree height (ß = 0.48, *p* < 0.001, *R* ^2^ = 0.2) and season (ß = 0.23, *p* = 0.01, *R* ^2^ = 0.07), whereas it was negatively affected by the presence of water bodies (ß = −0.25, *p* = 0.03, *R* ^2^ = 0.05). Similarly, the percentage of butterflies consuming lichens or algae during their larval stages was significantly affected by habitat traits (*p* < 0.001, *R* ^2^ = 0.58 ± 0.04) with a significant (and positive) affect from the factor tree height (ß = 0.63, *p* < 0.001, *R* ^2^ = 0.39) (Table 2).

**FIGURE 3:**
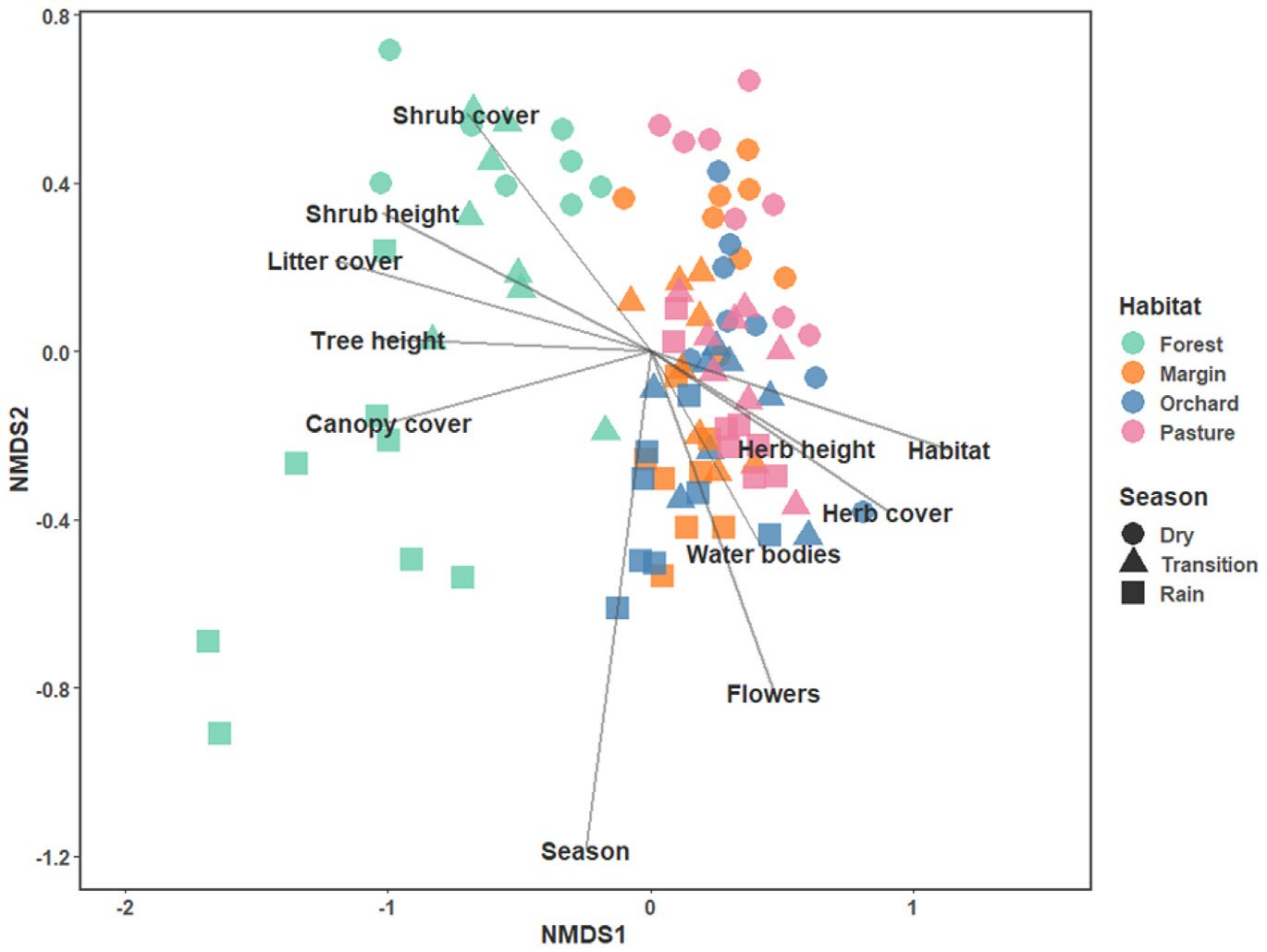
NMDS plot of butterfly communities in Kaya Kambe for all habitats and seasons, including environmental variables. Stress = 0.14.

An NMDS plot divided communities by both season and habitat (Figure 3). Rainy and dry seasons are distinct groups with the transition period being positioned between them but partly overlapping with the former. Overall, forest transects are separated from the three other habitat types, which are widely intermixed. PERMANOVA revealed significant differences in butterfly community composition among habitat–season combinations (Habitat × Season: *p* = 0.001, *R* ^2^ = 0.07), whereas no consistent main effects of habitat or season were detected. This indicates that seasonal changes in community structure were habitat‐specific. Butterfly community composition differed significantly among seasons within the forest (*p* = 0.001, *R* ^2^ = 0.31), its margins (*p* = 0.003, *R* ^2^ = 0.20), orchards (*p* = 0.002, *R* ^2^ = 0.19), and pastures (*p* = 0.002, *R* ^2^ = 0.23). Community composition was also shown to differ significantly among habitats within the dry season (*p* = 0.001, *R* ^2^ = 0.29), the transition period (*p* = 0.001, *R* ^2^ = 0.34), and the rainy season (*p* = 0.001, *R* ^2^ = 0.45). Additional plots for the three seasons all show the forest to be separated from the other habitat types along at least one axis. This separation is made especially clear in the results of a cluster analysis (Appendix S4).

A Mantel test found no significant correlation between the faunal matrix and the topographic distance matrix (r = −0.01, *p* = 0.52), indicating that spatial autocorrelation did not significantly influence the distribution of butterfly abundances.

### 3.3 Differences in Habitat Traits

**FIGURE 4:**
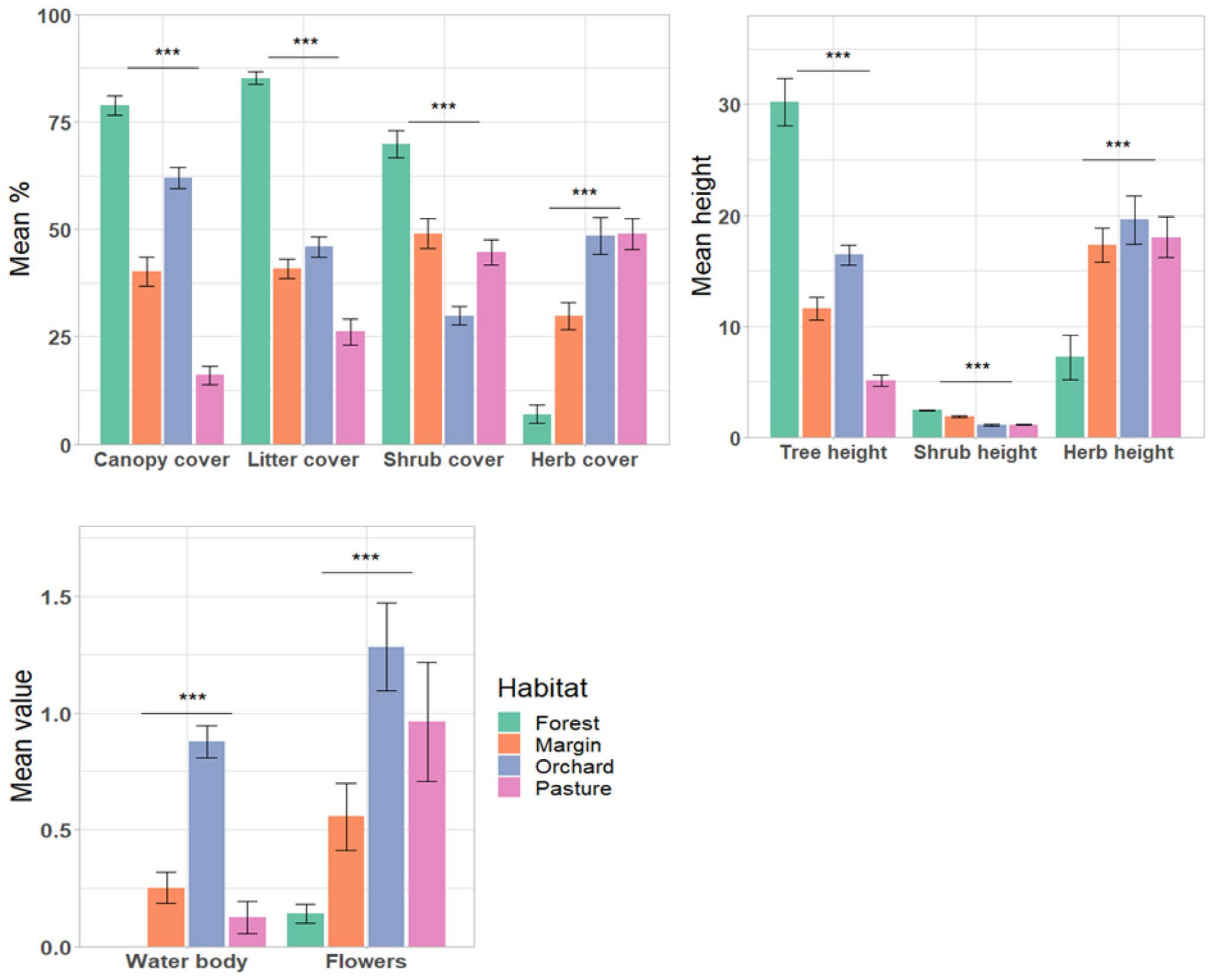
Mean habitat traits per transect. Shown are canopy cover, litter cover, shrub cover, and herb cover (top left), as well as tree height (in m), shrub height (in m), and herb height (in cm) (top right), as well as the presence of water bodies and number of flowers (below). Error bars denote conventional standard errors. Labels above groups refer to significance levels: NS *p* > 0.05, * *p* ≤ 0.01, **p ≤ 0.01, ***p ≤ 0.001.

**FIGURE 5:**
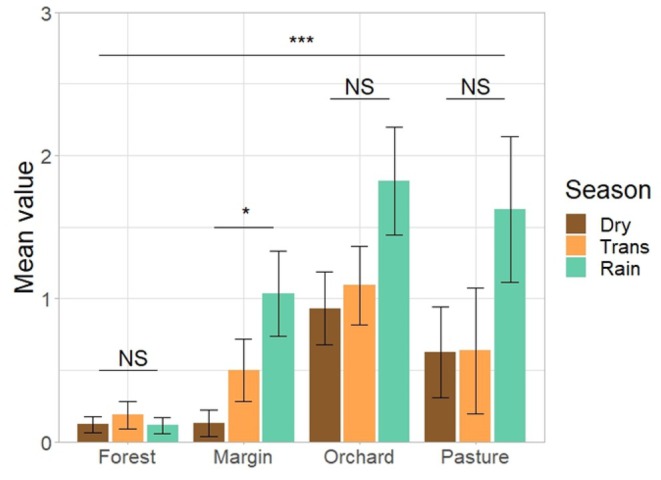
Mean values for the number of flowers across all habitats for each season. Label above group refers to significance level: NS *p* > 0.05, * *p* ≤ 0.01, **p ≤ 0.01, ***p ≤ 0.001.

Canopy cover (H(3) = 72.23, *p* < 0.001), tree height (H(3) = 66.45, *p* < 0.001), shrub cover (H(3) = 47.58, *p* < 0.001), shrub height (H(3) = 72.1, *p* < 0.001), herb cover (H(3) = 51.74, *p* < 0.001), herb height (H(3) = 22.17, *p* < 0.001), litter cover (H(3) = 65.8, *p* < 0.001), presence of water bodies (H(3) = 52.23, *p* < 0.001), and number of flowers (H(3) = 22.46, *p* < 0.001) differed significantly among habitats (Figure 4). Differences in the number of flowers were also significant when comparing seasons (H(2) = 8.32, *p* = 0.02) (Figure 5).

## 4 Discussion

### 4.1 Species Diversity and Abundance

Species richness and abundance differed significantly among the four habitat types (Table 1). Both were highest in orchards and lowest in the forest throughout our study period. These findings are congruent with previous observations in the tropics (Winfree et al. 2009; Kudavindanage et al. 2012; Schmitt et al. 2020). The most likely explanation is the availability of specific resources such as flowers and water bodies found in anthropogenic habitats throughout time, which strongly attract many butterfly species (Dennis et al. 2006). For example, the number of flowers was significantly higher in orchards compared with all other habitats throughout time (Figure 5). However, we also have to take into account that butterflies are less visible in forest ecosystems than in the other assessed ones; in addition, they scatter more along the third dimension because of the height of the trees in which they might even accumulate in the canopy (DeVries 1988). Consequently, the low count numbers in comparison with the other habitat types might only partly be real. Further studies are necessary for addressing this aspect in detail.

Species evenness was significantly higher in the forest than in all other habitat types. This is mainly due to the strong dominance (including mass migrations) of a few habitat generalists in anthropogenic landscapes, such as *Catopsilia florella*, *Junonia oenone*, and *Danaus chrysippus*. Such occurrences of individuals were not observed in the forest, where all species occurred in comparatively small numbers and were evenly distributed. Similar differences in the distribution of species have already been found in other studies on butterfly diversity in natural and anthropogenic habitats in the tropics (Sundufu and Dumbuya 2008; Washko 2017; Schmitt et al. 2020).

### 4.2 Community Structures

The butterfly community in the natural forest habitat differed significantly from the three anthropogenic habitat types (Figure 3). These differences in species composition apparently are directly related to the varying availabilities of specific resources and structures of the respective habitat types, demonstrated in our study. For example, the forest ecosystem contains considerably more typical forest butterfly species adapted to this shady and cooler habitat, also having their larval habitats there (see DeVries 1988; Larsen 1991, 2005; Bobo et al. 2006; Vasconcelos et al. 2015; Habel et al. 2018; Schmitt et al. 2020). In contrast, anthropogenic habitats are sunnier and more open. In consequence, they harbour many ecologically ubiquitous species found in open areas (Larsen 1991, 2005; Fermon et al. 2003; Van Vu 2008; Habel et al. 2018; Schmitt et al. 2020, 2021).

These contrasting conditions are reflected by the respective characteristics of the community assemblies. Thus, high hemeroby indices were mostly found for taxa in anthropogenic habitats (Table 1). Consequently, species mainly found in anthropogenically disturbed landscapes are often widespread generalist species, which cope well with a broad variety of ecosystems and resources (cf. Manne and Pimm 2001; Kumar et al. 2018; Behroozian et al. 2020). This conclusion is supported by the fact that the geographical distribution of butterflies in the study area was found to be highly positively correlated to their tolerance of anthropogenic influences. On the other side, *Baliochila minima*, the single Kenyan endemic butterfly species found in our study area, mostly exclusively occurred in the forest interior. In general, six out of the 12 species that do not cope with anthropogenic disturbance were mainly or even exclusively found inside the forest and along its margins (i.e., *Acraea rabbaiae, Baliochila minima, Coeliades libeon, Euxanthe wakefieldii, Teriomima micra, and T. subpunctata*). Only three out of these species (*Axiocerses punicea, Graphium kirbyi, and Lepidochrysops lukenia*) were observed in orchards and pastures. Similar patterns have already been demonstrated for butterflies in previous studies from the tropics (Bobo et al. 2006; Vasconcelos et al. 2015).

Although interpreting these differences among butterfly communities among the four habitat types, it has to be considered that we applied a sampling method, which mostly covers occurrences of butterflies near the ground. However, as already mentioned above, a significant proportion of butterflies in tropical forests occurs in the canopy (DeVries 1988; Fermon et al. 2003; Molleman et al. 2006). Hence, the diversity in the forest ecosystem is likely underestimated in our study. This, however, should not conflict with its distinctiveness from the anthropogenic habitats.

Considering the expected increase in specialisation towards more natural habitats, it is surprising that the mean larval diet breadth was found to be significantly higher for taxa found mainly in the forest than in all other habitat types (Table 1). An increase in larval diet breadth from natural to anthropogenic habitats was also observed in a previous study on butterflies from southern Kenya (Schmitt et al. 2020). However, butterfly larvae feeding exclusively on lichens or algae could not be included in this calculation on the specialisation of phagy for the present study, given that the exact species and genera they feed on are largely unknown until today. However, several species with high importance for the forest butterfly community (i.e., *Baliochila hildegarda, B* *. minima* , *Teriomima micra, and T. subpunctata*) belong to this group of larval food specialisation. Furthermore, the lack of sampling in higher vegetation strata may have led to some specialised butterfly species being missed (see above).

### 4.3 Effects of Seasonality

Species richness, abundance, and community diversity were highest during the rainy season and lowest during the dry season (Tables 1 and 2). This is well reflected in the individual species. Thus, some species (e.g., *Melanitis leda* and *Tagiades flesus*; Appendix S4) were not or very rarely observed during the dry season but began appearing in large numbers at the end of the transition period. Others experienced temporary abundance peaks during the transition period (e.g., *Danaus chrysippus, Andronymus neander*; Appendix S4) or early rainy season (e.g., *Graphium antheus*; Appendix S4). However, a limited number of species even showed opposite trends with decreasing abundances from dry to rainy season (e.g., *Byblia ilithyia, Colotis evagore, Eurema hecabe, Baliochila minima*; Appendix S4).

Such findings in general correspond with previous studies on butterfly communities in tropical regions (Grøtan et al. 2012, 2014; Habel et al. 2018; Schmitt et al. 2020, 2021). Resource availability and thus living conditions in general are much improved during the rainy season, which therefore causes the boost of insect populations at its very beginning, that is, with the onset of the transition period (Kishimoto‐Yamada and Itioka 2015). As a consequence, life cycles of many insects correlate with seasonality, also in the tropics, and thus many butterfly species eclose (or break their diapause) with the onset of the rains (H. Wolda 1988; Henk Wolda 1989; Lourenço et al. 2020). In our case, mass eclosion of generalist butterfly species such as *Hypolimnas misippus* and *Danaus chrysippus* with the first heavy rainfalls was largely responsible for the strong increase in abundance.

Furthermore, low resource availability in the dry season is known to cause movements over larger distances and thus enhance intermixture, making communities of different habitat types more similar (see Grøtan et al. 2012, 2014; Habel et al. 2018). In turn, differentiation among butterfly communities is increasing among distinct habitat types during the rainy season. These community fluctuations in general are even more pronounced in natural forest habitats, and less in anthropogenic habitats, as also revealed in our data (Figure 3). Hence, fluctuations of environmental conditions might be buffered by human activities (planting of vegetation, gardens, water availability), as already shown in previous studies on butterflies and other pollinating insects in Africa (Norfolk et al. 2014; Habel et al. 2018; Schmitt et al. 2021). Corresponding with these findings, none of the functional traits was significantly different among seasons within anthropogenic habitats like orchards and pastures (Table 2), indicating that functional communities within these habitats barely changed along the course of the study period. In turn, some traits corresponding with living in forests changed significantly among seasons (Table 2), with the most typical forest communities obtained during the rainy season (Figure 3) when no individuals of the typical open land species take refuge in the shadier forest habitats.

### 4.4 Relevance of Natural Forest and Surrogate Habitats

We recorded individuals of some forest‐dependent species in other habitat types such as forest‐like orchards (e.g., *Euphaedra neophron*, *Tagiades flesus*, *Graphium kirbyi*, and *Neptis carcassoni*). This indicates that orchards, to some extent, can be surrogate habitats for forest species. However, the entire life cycle of a butterfly must be considered and underlines the high ecological relevance of still intact natural forest for successful development and thus the existence of typical forest butterfly species. Most of the forest specialist butterflies depend on specific larval food plants for larvae and adults, as well as specific microhabitat conditions (e.g., Habel et al. 2018). Therefore, these species might be occasional visitors of surrogate habitats, but their successful development from larvae to the adult stage is only possible in intact natural forests, where the specific larval food plants are growing and where specific microclimatic conditions exist (cf. Munyuli 2012; Winfree et al. 2011; Habel et al. 2018). As a general consequence, the conservation of the last natural forest fragments along the coast of East Africa is therefore of pivotal importance for nature conservation.

### 4.5 Study Limitations

Our study area is spatially restricted to a limited geographic area (one single forest patch). However, many species can only survive in the long term if there are other suitable habitats in close geographic proximity, thus enabling the exchange of individuals and subsequently the stability of populations and species (Hanski 1991). Numerous studies have shown that small forest fragments can only sustain species in the long term to a limited extent (see Laurance et al. 2011, 2018). In the case of Kaya Kambe, the next forest remnants are more than 3 km away, and less than 20 other forest fragments exist in a radius of 30 km. It would be of high interest to analyse population and species viability at the landscape level (incorporating a large number of single forest fragments scattered across the landscape). However, this was not the main focus of our study on biodiversity within a habitat mosaic.

Even within the already studied area, further studies with complementary methodologies would be beneficial. It is well‐known that assessing butterfly assemblages in tropical forests solely on the basis of Pollard transect walks at ground level has at least two shortcomings: the risk of missing canopy species (or generally of smaller‐sized species difficult to spot inside a forest, like many lycaenids and hesperids), and the risk of under‐sampling the guild of rotting‐fruit feeders (that are better monitored using baited traps) (see Aduse‐Poku et al. 2012; Ribeiro et al. 2015; Checa et al. 2018). In addition, our transects in the forests and at their margins were placed quite close to each other (often not more than 100 m distant), as the geographic extent of these habitat types was quite small. Although the effect of spatial autocorrelation on butterfly abundance was shown to be largely negligible in our study, large and mobile butterfly species might still spatially intermix at least to some extent. This might lead to some pseudo‐replication so that the affected transects might not be completely independent. Therefore, supplementary data collection with larger distances among transects, including further forest fragments, would be desirable.

## Author Contributions

**Laura Wagner:** data curation (equal), formal analysis (equal), methodology (equal), software (equal), validation (equal), visualization (equal), writing – original draft (equal), writing – review and editing (equal). **Jan Christian Habel:** conceptualization (equal), investigation (equal), methodology (equal), project administration (equal), supervision (equal), writing – original draft (equal), writing – review and editing (equal). **Maria Fungomeli:** data curation (equal), investigation (equal), writing – original draft (equal). **Mike Teucher:** data curation (equal), funding acquisition (equal), writing – original draft (equal), writing – review and editing (equal). **Thomas Schmitt:** conceptualization (equal), data curation (equal), formal analysis (equal), funding acquisition (equal), investigation (equal), methodology (equal), project administration (equal), resources (equal), software (equal), supervision (equal), validation (equal), visualization (equal), writing – original draft (equal), writing – review and editing (equal).

## Funding

This work was supported by German Academic Exchange Service London, Biodiversitätsnetzwerk ‐ Biocult.

## Conflicts of Interest

The authors declare no conflicts of interest.

## Supporting information


**Appendix S1:** ece373242‐sup‐0001‐Appendix1.xlsx.

**Appendix S2:** ece373242‐sup‐0002‐Appendix2.docx.

**Appendix S3:** ece373242‐sup‐0003‐Appendix3.docx.

**Appendix S4:** ece373242‐sup‐0004‐Appendix4.docx.

**Appendix S5:** ece373242‐sup‐0005‐Appendix5.docx.

## Data Availability

All raw data used in this study are available as Supporting information in electronic appendices.
