# Supplementary material for: Butterfly Diversity in a Sacred Kaya Forest in Southern Kenya
Source: Ecol Evol. 2026 Mar 20;16(3):e73242. doi: 10.1002/ece3.73242 (PMC13093687; doi:10.1002/ece3.73242)
Supplement: Supplementary file 2 — Appendix S2: ece373242‐sup‐0002‐Appendix2.docx. [file ECE3-16-e73242-s001.docx]

**Appendix 2**

**BUTTERFLY TRAITS**

**Distribution**: 1 = Kenyan endemic, 2 = endemic to East Africa, 3 = restricted to eastern and southern Africa (including the Arabian Peninsula), 4 = restricted to sub-Saharan Africa (including western Africa), 5 = distributed across the African continent and beyond.

**Phagy of larvae**: 1 = monophagous (restricted to one food plant genus), 2 = oligophagous (restricted to one food plant family), 3 = polyphagous (more than one food plant family).

**Larval foodplant type**: 1 = mainly grasses and herbs, 2 = herbaceous and woody plants in mostly equal parts, 3 = mainly bushes and trees.

**Larvae feeding on lichens/algae**: 0 = no, 1 = yes.

**Hemeroby**: 1 = only natural habitats, 2 = accepting disturbed natural habitats, 3 = occurring in natural and anthropogenic habitats, 4 = more common in anthropogenic than in natural habitats.

**Water dependency**: 1 = occurring in truly arid habitats, 2 = accepting intermediate arid habitats, 3 = not occurring in arid habitats.

**Savannah index**: 1 = not occurring in savannahs, 2 = mainly outside savannahs, 3 = in savannahs and forests, 4 = mainly in savannahs, 5 = only in savannahs.

**Tree dependency**: 1 = not requiring trees, 2 = requiring trees, 3 = exclusively in forests.

Classifications were adopted from Larsen (1991), Schmitt et al. (2020, 2021), and online data bases (metamorphosis.org.za and learnaboutbutterflies.com).
