# Supplementary material for: Butterfly Diversity in a Sacred Kaya Forest in Southern Kenya
Source: Ecol Evol. 2026 Mar 20;16(3):e73242. doi: 10.1002/ece3.73242 (PMC13093687; doi:10.1002/ece3.73242)
Supplement: Supplementary file 3 — Appendix S3: ece373242‐sup‐0003‐Appendix3.docx. [file ECE3-16-e73242-s002.docx]

Appendix 3:

**STATISTICAL ANALYSES**

**Temporal development of species abundances and dominance** ‒ To investigate the influence of seasonal changes on species’ abundances, the total abundance of each species was calculated over all transects and the percentage during each sampling round. Similarly, species' dominance (i.e. proportional share of total butterfly abundance) was calculated for each sampling round. We used ggplot() function to plot the development of species abundance and dominance for the 26 most common species (i.e. all species > 30 total records). Graph were smoothed using the smoothing method LOESS ("Locally Estimated Scatterplot Smoothing") to make patterns more easily visible.

**Diversity indices** ‒ Shannon indices (on the basis of natural logarithm) were obtained using diversity() in package "vegan" (Oksanen et al., 2022). Species evenness was calculated for each transect per season by dividing Shannon index with the natural logarithm of the respective total species number (i.e. the highest possible Shannon value). Inverted Simpson indices were calculated using alpha.div() function in package "asbio" (Aho, 2022).

**Species traits and environmental parameters** ‒ We estimated the mean values of all species traits for each transect and season. Calculations were based on log-transformed abundance data to prevent disproportional influence of highly abundant species.

**Comparisons of means** ‒ Kruskal-Wallis *H* tests were performed to compare mean species richness per sampling round, abundance, Shannon index, inverted Simpson index and Evenness among seasons and habitats using "stats" package. We performed four types of comparisons for each variable, i.e. (i) among all habitats across the entire period, (ii) among all seasons across all habitats, (iii) among all habitats within each of the seasons, and (iv) among all seasons within each of the habitats. Kruskal-Wallis *H* tests with significant results were then followed by pairwise Wilcoxon rank sum tests to obtain pairwise comparisons using pairwise.wilcox.test() in "stats". These two tests were also performed to compare habitat parameters and species traits among habitats. Species traits were additionally compared among the three seasons and within each season and habitat.

**Linear models** ‒ Linear regression models were used to assess the influence of habitat traits, habitat type, and season on abundance, species richness, diversity indices, and species traits. To avoid multicollinearity, variance inflation factor (VIF) was calculate for each predictor variable (i.e. canopy cover, litter cover, and habitat type) and subsequently predictors with a VIF > 5 were removed (Fox & Weisberg, 2019). Pearson correlation coefficients for the dependent variables were computed and if multiple variables were correlated by more than r = 0.7, all but one of them were removed from the model. Consequently, the variables Shannon index, Evenness, geographic distribution, larval food plant type, hemeroby index, water index, and savannah index were excluded. Six individual linear regression models were performed using "stats" package. Data were Box Cox transformed using "MASS" (Venables & Ripley, 2002) whenever a violation of the linear regression assumptions was indicated, i.e. for abundance, larval diet breadth, consumption of lichens/algae, and tree index. As consumption of lichen/algae was a binary trait and the number of species found to consume this larval food type was low, clear violation of the regression assumptions was detected; Box Cox transformation could only lightly correct this violation.

**NMDS** ‒ Non-metric multidimensional scaling (NMDS) was performed to compare butterfly species composition across habitats and seasons. Analysis was performed for the entire study period and for each of the seasons separately. Abundances were log(x+1)-transformed before analysis. We utilized metaMDS() function in “vegan” package (Oksanen et al. 2020). Bray-Curtis dissimilarities were applied and the number of dimensions was set to k = 3 to reduce stress.

**Cluster analysis** ‒ Hierarchical clustering was performed on the log(x+1)-transformed original 96 transect x 94 species and the 32 transect x 94 species tables using hclust() function. The optimal number of clusters was determined with n_clusters() in "parameters" (Lüdecke et al., 2020). The number of clusters was set to 2 as supported by the majority of methods (43.8 % for the original and 27.3 % for the merged community table). Dendrograms were created with as.dendrogram() in "stats", cutree() in "dendextend" (Galili, 2015), dendrapply() in "stats", and plot() in "base". We created scatterplots with fviz_cluster() in "factoextra" (Kassambara & Mundt, 2020).
