# Supplementary material for: Butterfly Diversity in a Sacred Kaya Forest in Southern Kenya
Source: Ecol Evol. 2026 Mar 20;16(3):e73242. doi: 10.1002/ece3.73242 (PMC13093687; doi:10.1002/ece3.73242)

**Appendix 4**

| **Traits** | **All habitats** | **Forest** | **Forest margin** | **Orchard** | **Pasture** |
| --- | --- | --- | --- | --- | --- |
| **Canopy cover (%)** | 49.2 (± 2.7) | 78.8 (± 2.3) | 40.2 (± 3.4) | 61.9 (± 2.5) | 16.0 (± 2.1) |
| **Litter cover (%)** | 49.5 (± 2.5) | 85.2 (± 1.5) | 40.8 (± 2.3) | 45.9 (± 2.4) | 26.2 (± 3.1) |
| **Shrub cover (%)** | 48.3 (± 2.1) | 69.8 (± 3.2) | 49.0 (± 3.5) | 29.9 (± 2.1) | 44.6 (± 3.0) |
| **Herb cover (%)** | 33.6 (± 2.4) | 7.0 (± 2.1) | 29.8 (± 3.2) | 48.5 (± 4.2) | 49.0 (± 3.6) |
| **Tree height (m)** | 15.8 (± 1.1) | 30.2 (± 2.2) | 11.6 (± 1.0) | 16.4 (± 0.9) | 5.1 (± 0.5) |
| **Shrub height (m)** | 1.6 (± 0.1) | 2.4 (± 0.1) | 1.9 (± 0.1) | 1.1 (± 0.1) | 1.1 (± 0.0) |
| **Herb height (cm)** | 15.5 (± 1.1) | 7.2 (± 2.0) | 17.3 (± 1.6) | 19.6 (± 2.2) | 18.0 (1.8) |
| **Water body** | 0.31 (± 0.05) | 0.00 (± 0.00) | 0.25 (± 0.07) | 0.88 (± 0.07) | 0.13 (± 0.07) |
| **Flowers** | 0.74 (± 0.10) | 0.14 (± 0.04) | 0.56 (± 0.14) | 1.28 (± 0.19) | 0.96 (± 0.26) |
| **Flowers_Dry_** | 0.45 (± 0.12) | 0.12 (± 0.06) | 0.13 (± 0.09) | 0.93 (± 0.25) | 0.63 (± 0.32) |
| **Flowers_Trans_** | 0.60 (± 0.15) | 0.19 (± 0.10) | 0.50 (± 0.22) | 1.09 (± 0.27) | 0.64 (± 0.44) |
| **Flowers_Rain_** | 1.15 (± 0.20) | 0.11 (± 0.06) | 1.04 (± 0.30) | 1.82 (± 0.38) | 1.63 (± 0.51) |

**Table A4-1: Mean habitat traits for all transects and separated by habitat type with conventional standard errors.** The number of flowers is additionally separated into dry season (Flowers_Dry_), transition period (Flowers_Trans_) and rainy season (Flowers_Rain_).

**Figure A4-1:** Total daily precipitation (mm) and mean temperature in the field (°C) measured during each of the 20 sampling rounds.


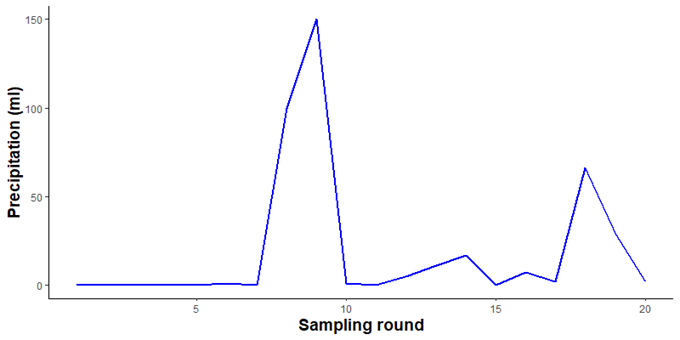


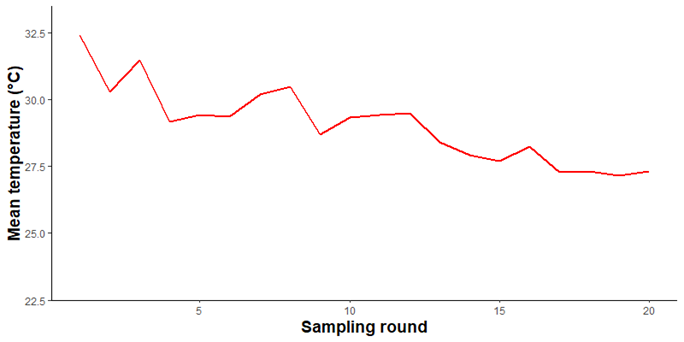


**Figure A4-2:** Mean species richness per sampling (a), and mean abundance (b), Evenness (c), Shannon index (d) and Simpson index (e) per transect for each habitat within each season. Error bars denote conventional standard errors. Labels above groups refer to significance levels: NS *P* > 0.05, * *P*≤ 0.01, ** *P* ≤ 0.01, *** *P* ≤ 0.001.


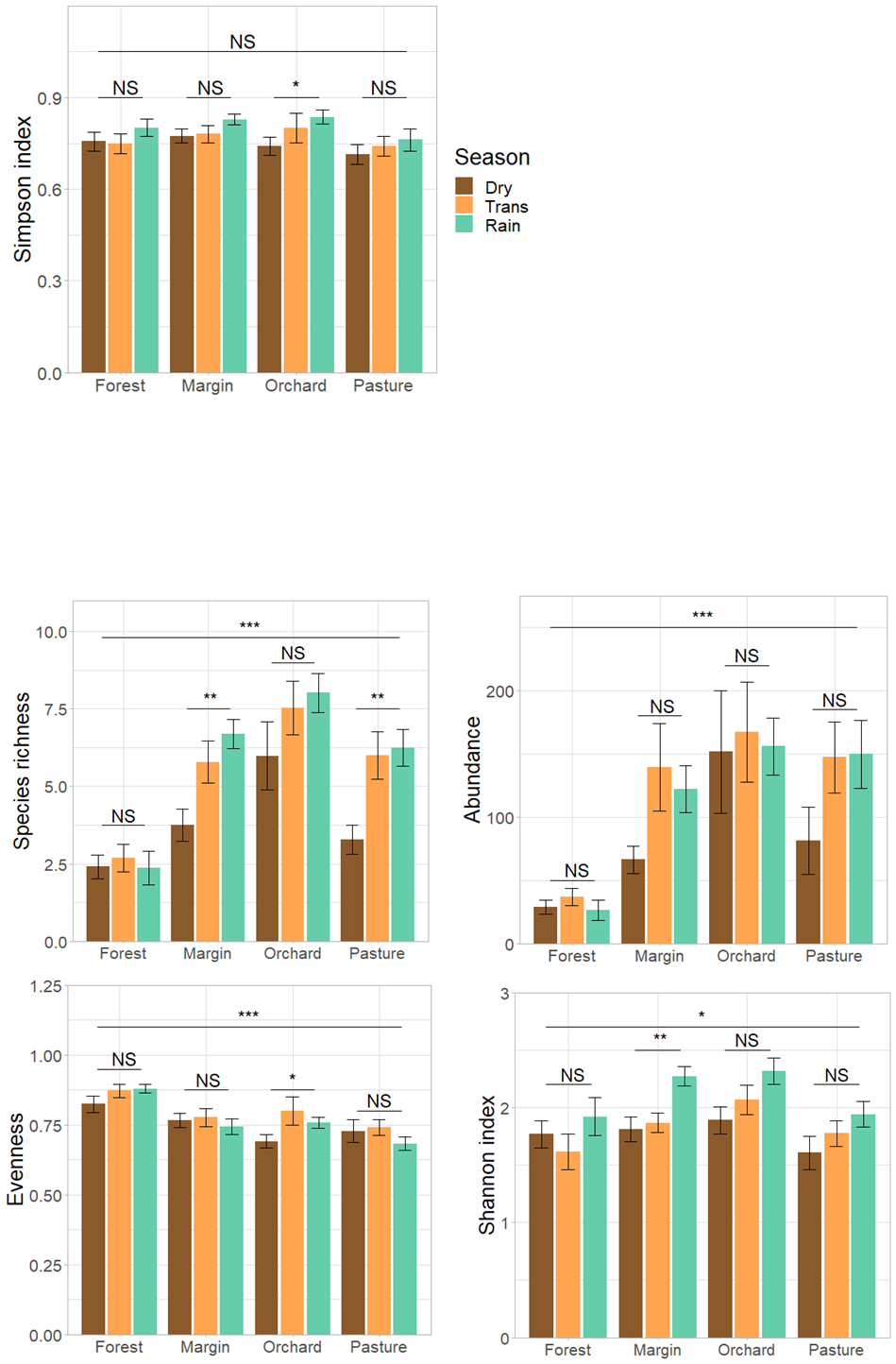

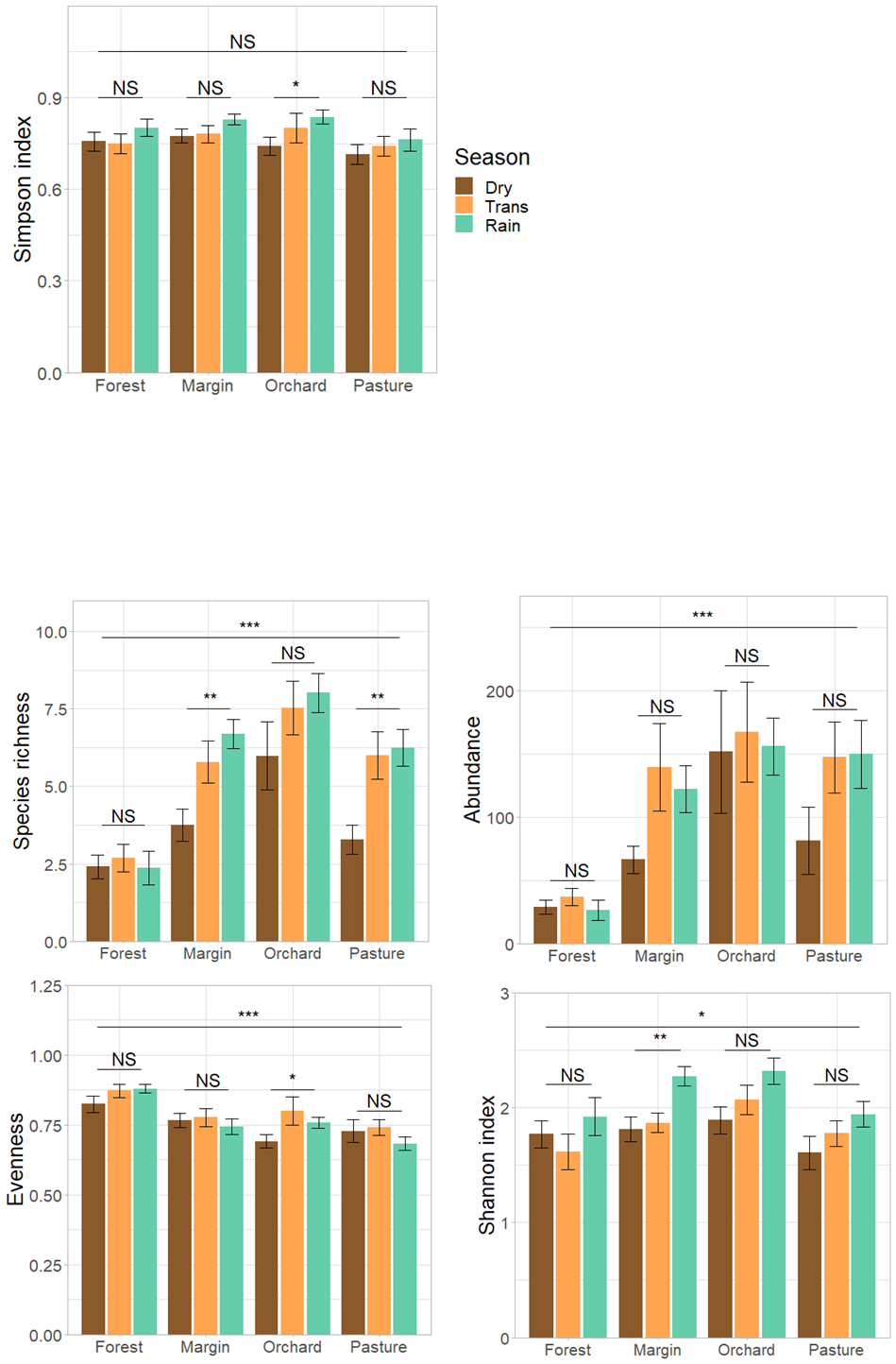


**Figure A4-3:** Temporal development of the abundances of 20 butterfly species showing different phenologies. (a) *Graphium antheus, Tagiades flesus, Melanitis leda, Junonia hierta*; (b) *Byblia ilithyia, Colotis evagore, Eurema brigitta,* *Baliochila minima*; (c) *Junonia oenone, Belenois thysa, Eurythela dryope, Baliochila hildegarda*; (d) *Danaus chrysippus, Hypolimnas misippus, Andronymus neander*, *Bicyclus safitza*; (e) *Junonia natalica, Colotis euippe, Euphaedra neophron*, *Belenois creona*.

(a)


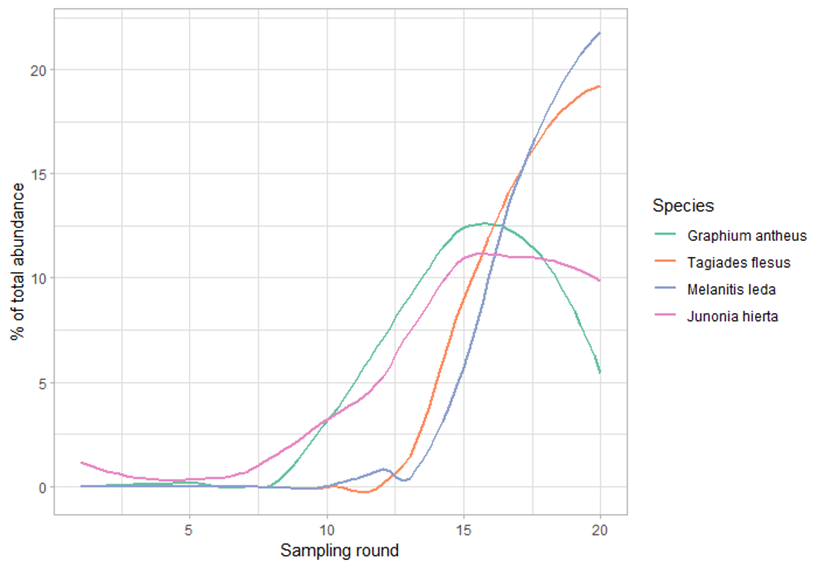


continued

(b)


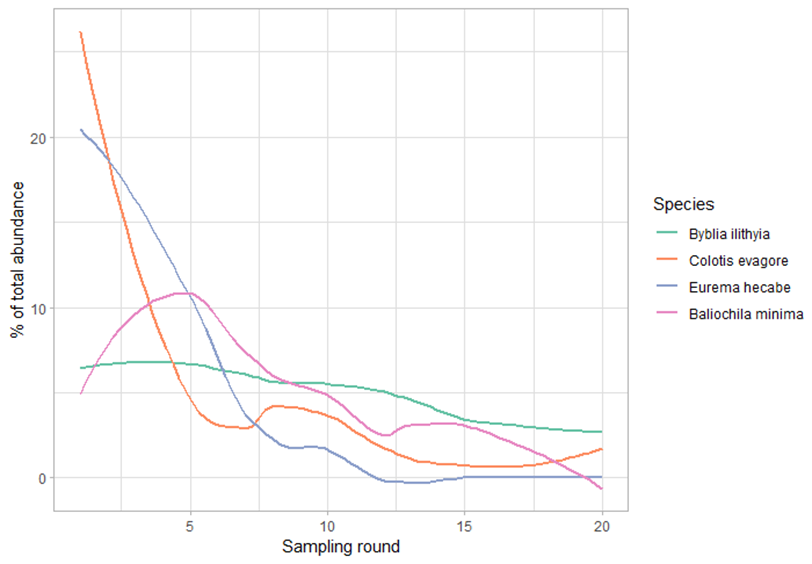


(c)


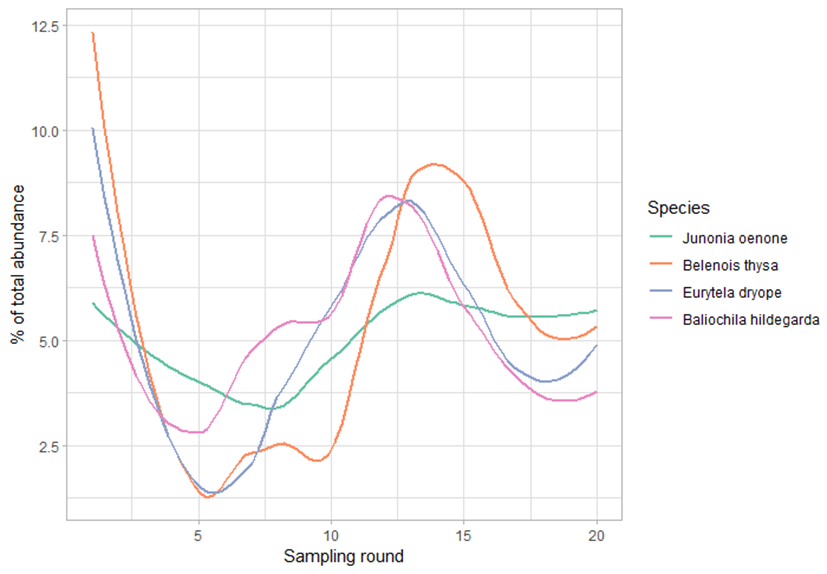


continued

(d)


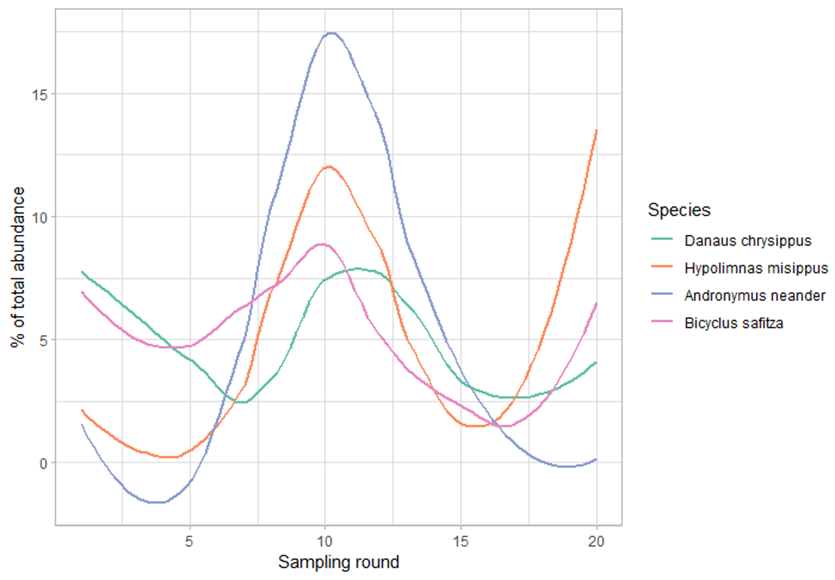


(e)


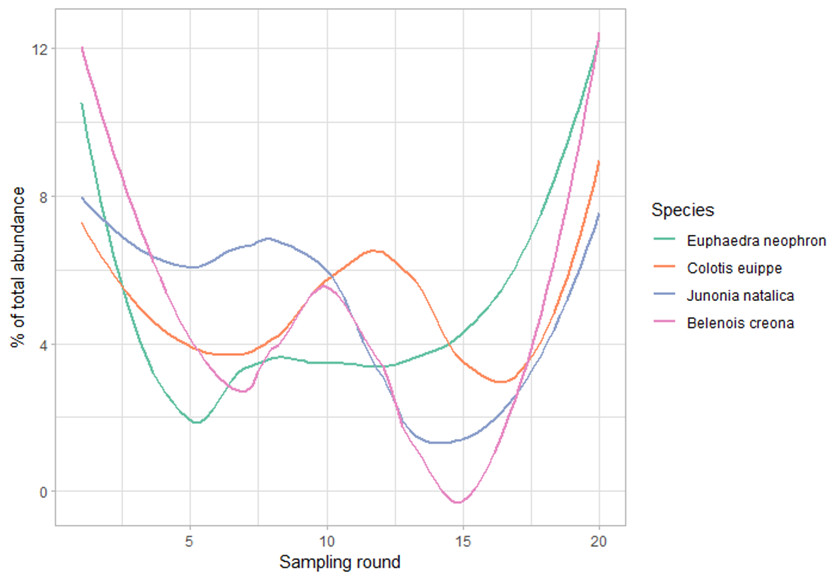


**Figure A4-4:** Mean values for distribution (a), larval diet breadth (b), larval food plant type (c) and lichen/algae consumption (d) for each habitat and season. Error bars denote conventional standard errors. Labels above groups refer to significance levels: NS *P* > 0.05, * *P* ≤ 0.01, ** *P* ≤ 0.01, *** *P* ≤ 0.001.


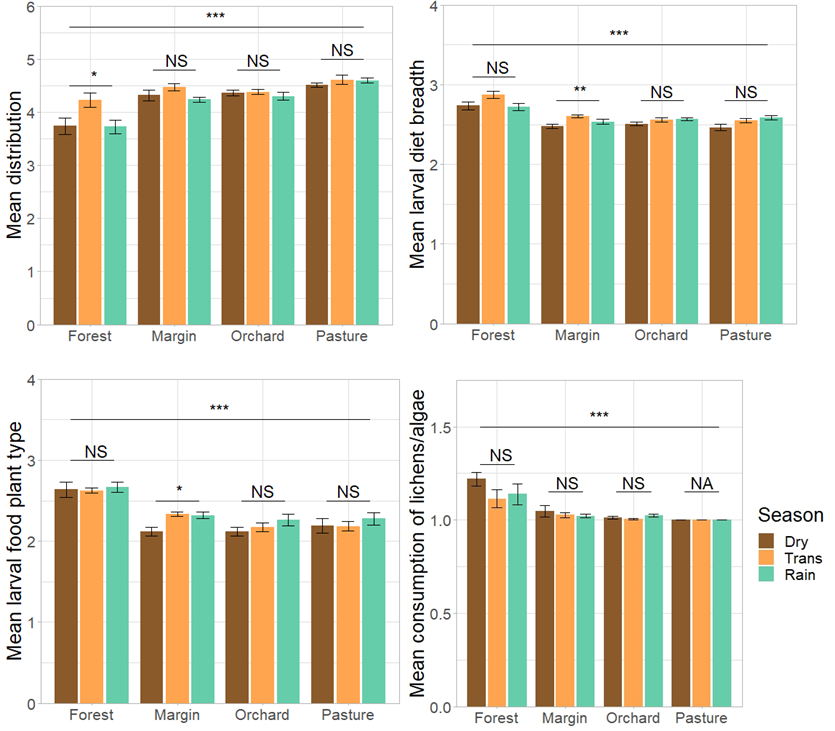


**Figure A4-5:** Mean hemeroby index (a), savannah index (b), water index (c) and tree index (d) for each habitat and season. Error bars denote conventional standard errors. Labels above groups refer to significance levels: NS *P* > 0.05, * *P* ≤ 0.01, ** *P* ≤ 0.01, *** *P* ≤ 0.001.


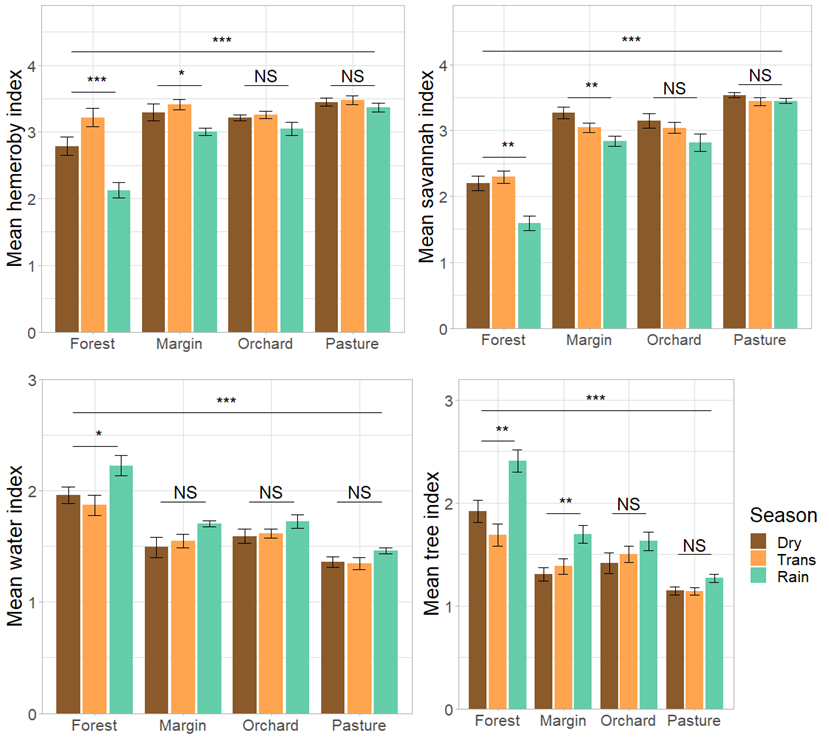


**Figure A4-6:** Scatterplot (a) and cluster dendrogram (b) of the butterfly communities of the 32 transects within the four habitat types (i.e. forest (Fo), forest margin (Ma), orchard (Or), and pasture (Pa)). The transects were automatically sorted into clusters 1 (green) and 2 (orange) based on similarities. For better clarity, the effect of seasons was excluded in these figures.


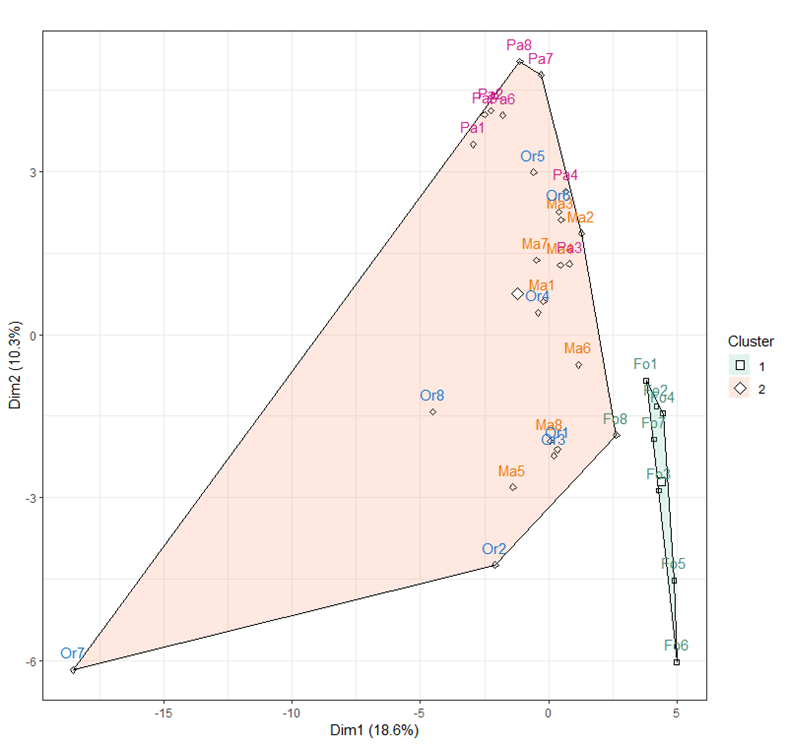


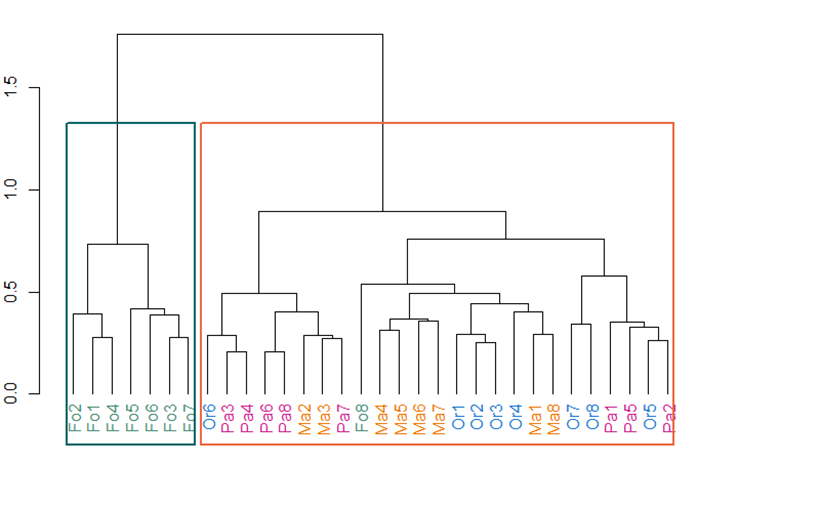

Supplement: Supplementary file 4 — Appendix S4: ece373242‐sup‐0004‐Appendix4.docx. [file ECE3-16-e73242-s003.docx]
