# Supplementary material for: Butterfly Diversity in a Sacred Kaya Forest in Southern Kenya
Source: Ecol Evol. 2026 Mar 20;16(3):e73242. doi: 10.1002/ece3.73242 (PMC13093687; doi:10.1002/ece3.73242)

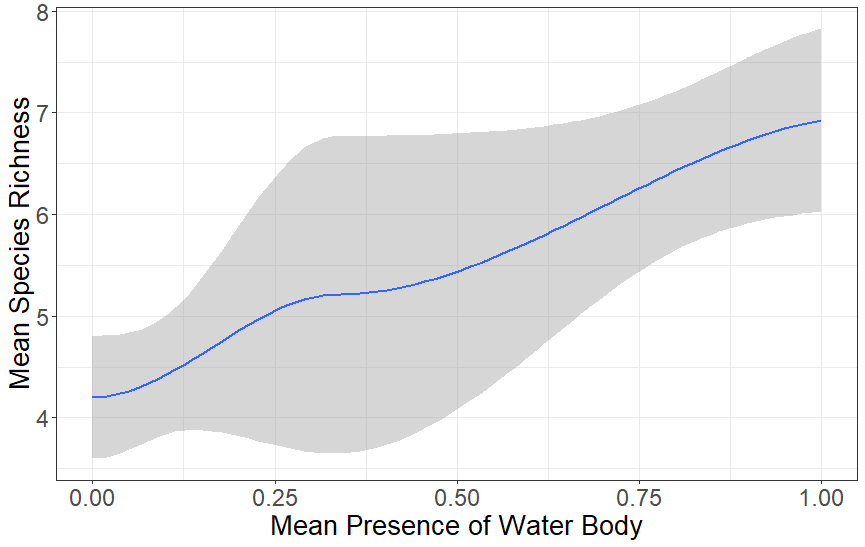

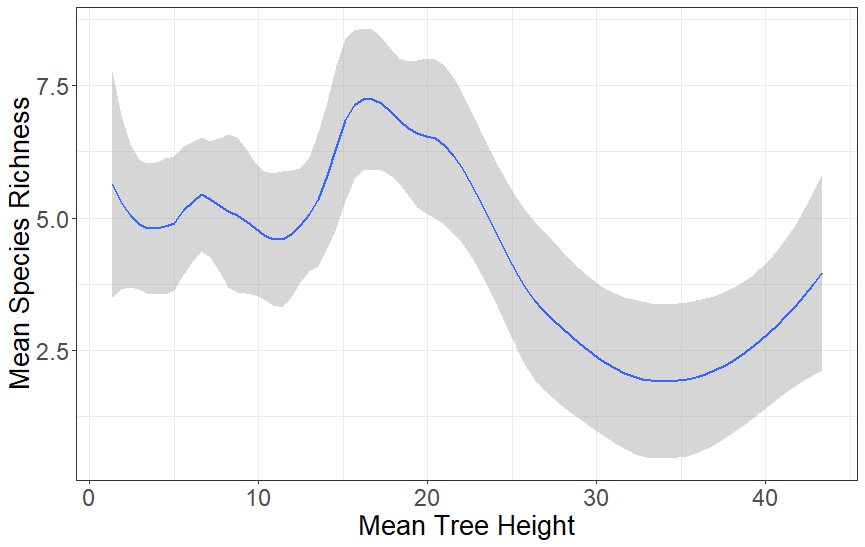

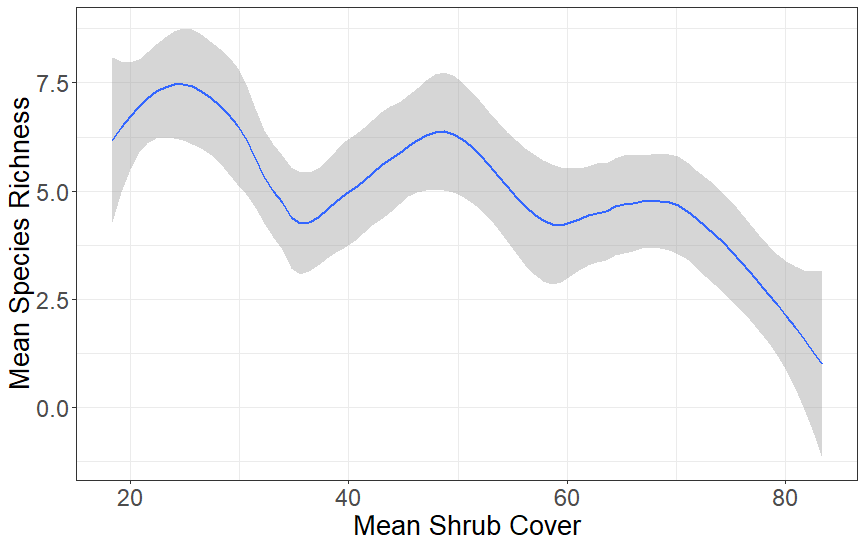

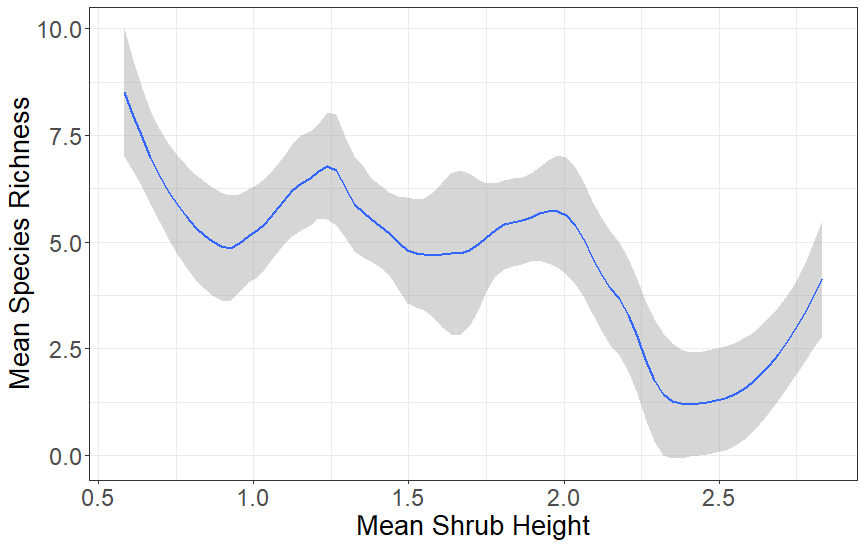
**
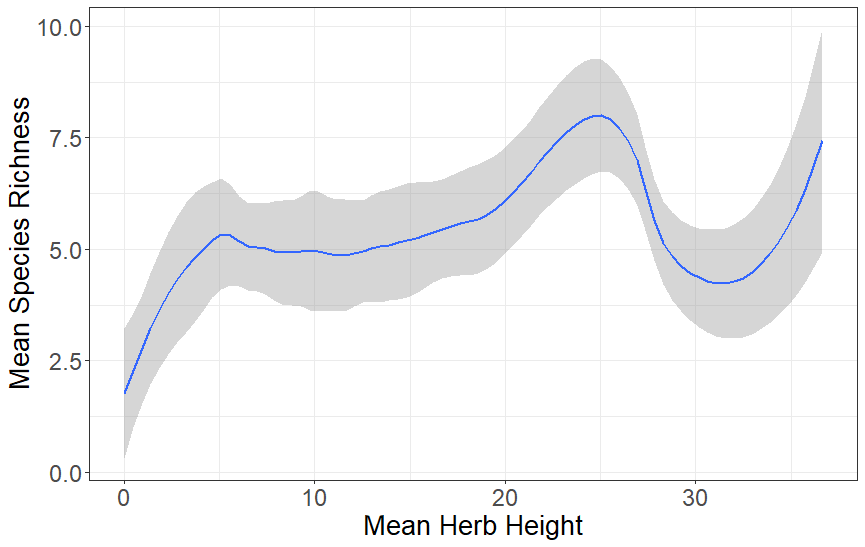

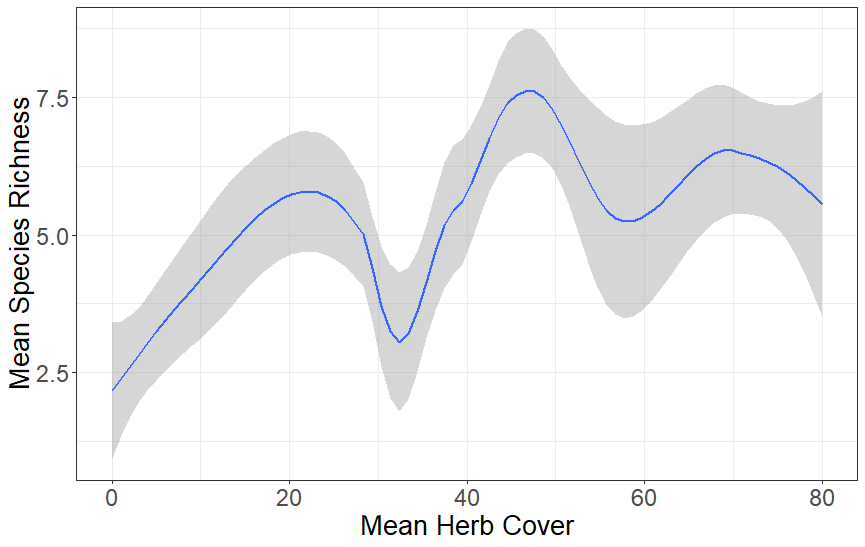
**
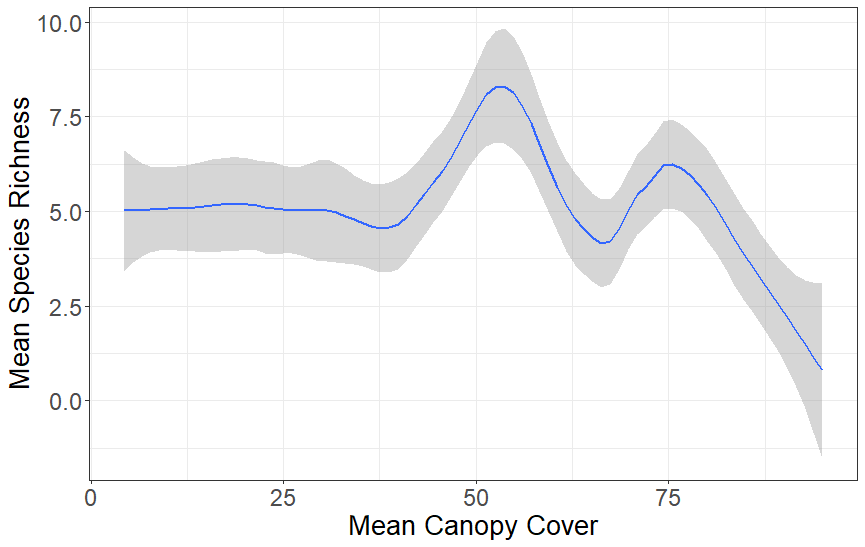

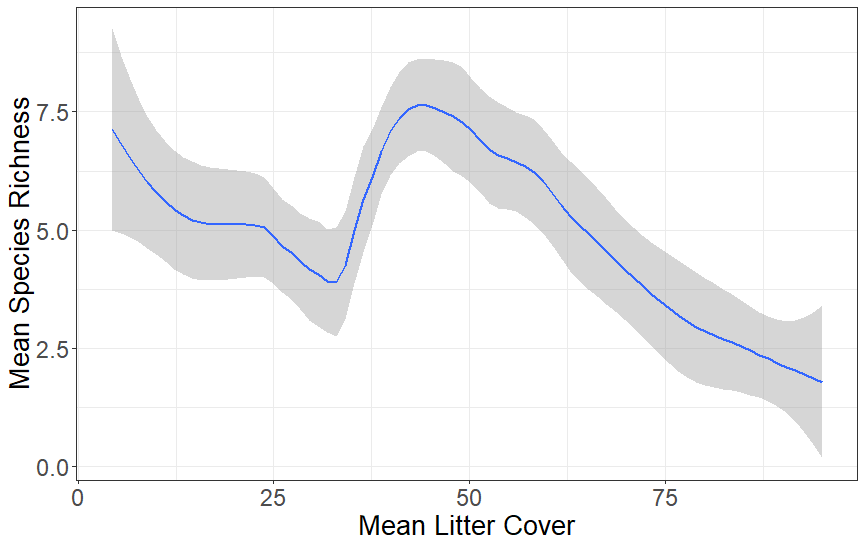
**Figure A5-1:** Line graphs of the relationships between species richness and habitat traits.


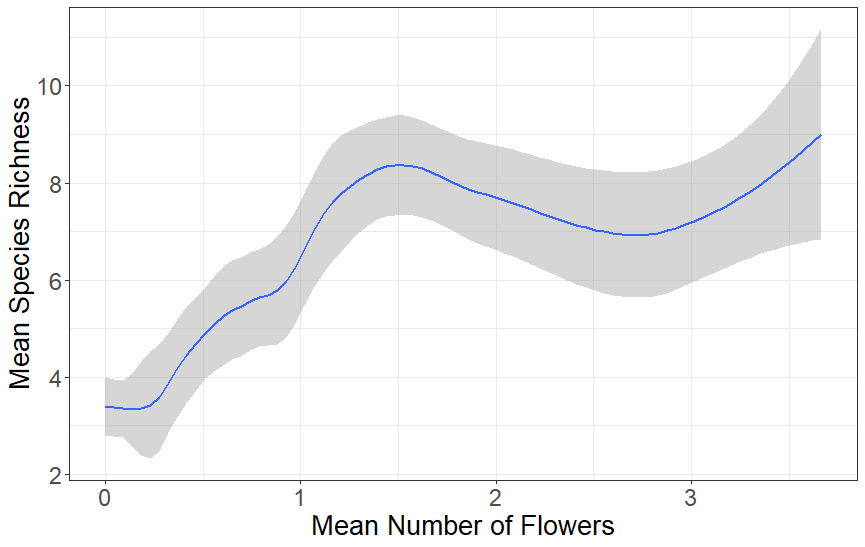


**Figure A5-2:** Line graphs of relationships between abundance and habitat traits.


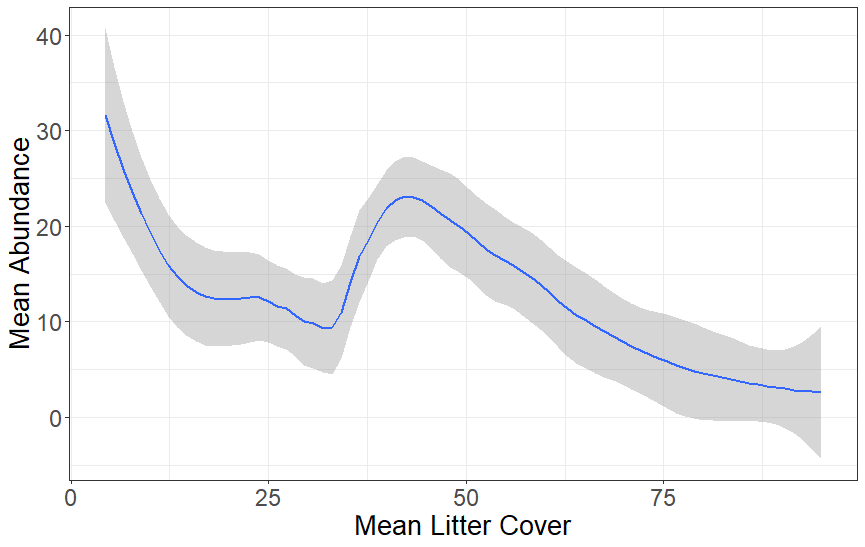

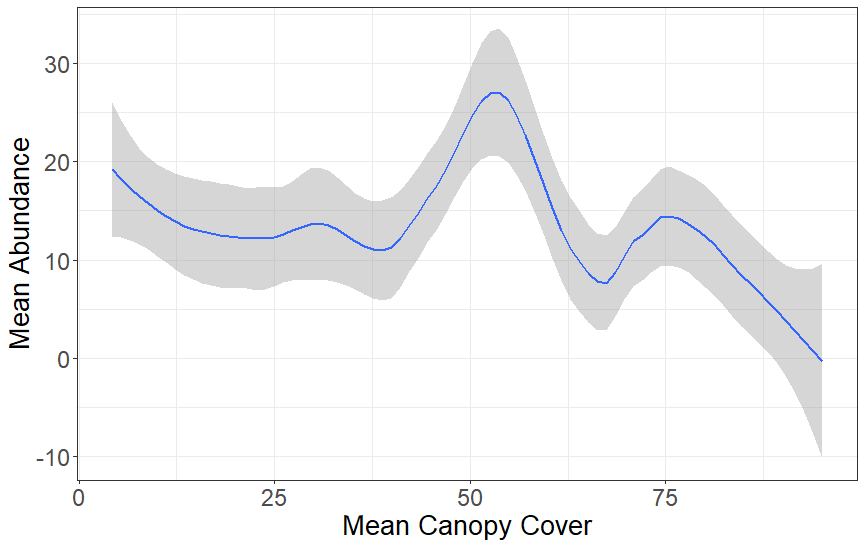

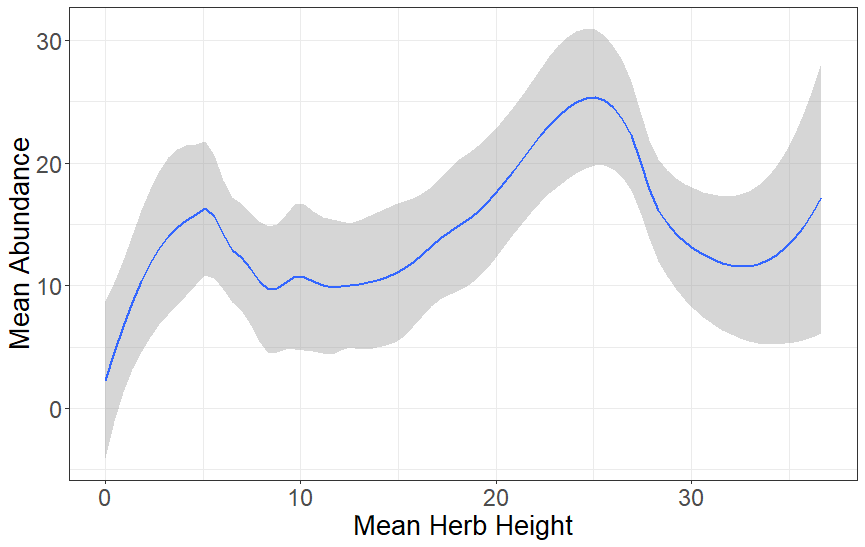

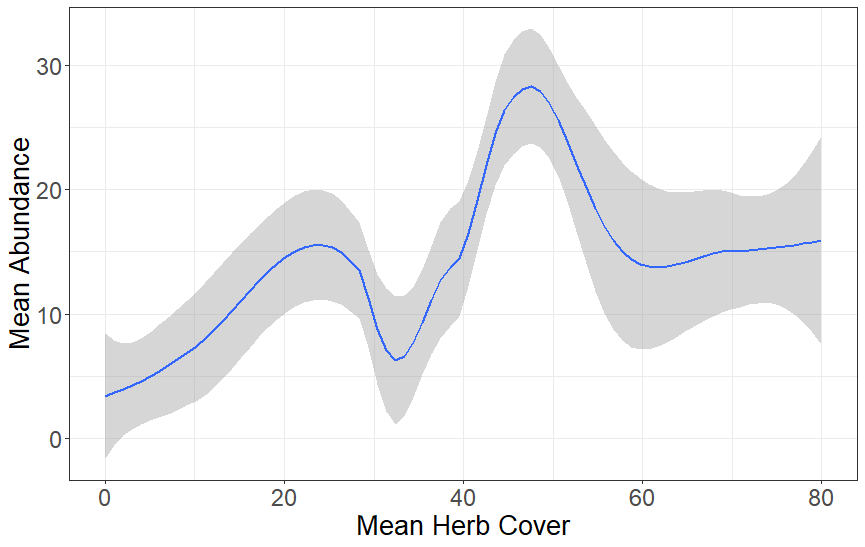

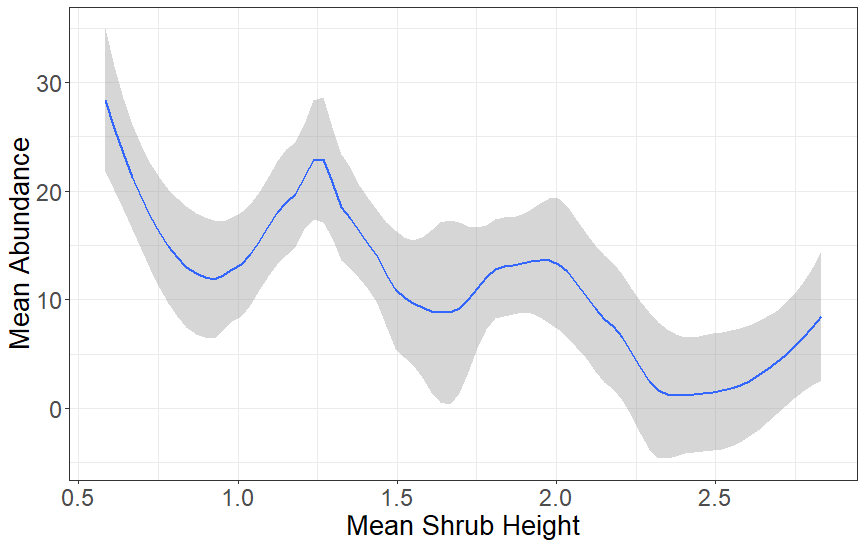

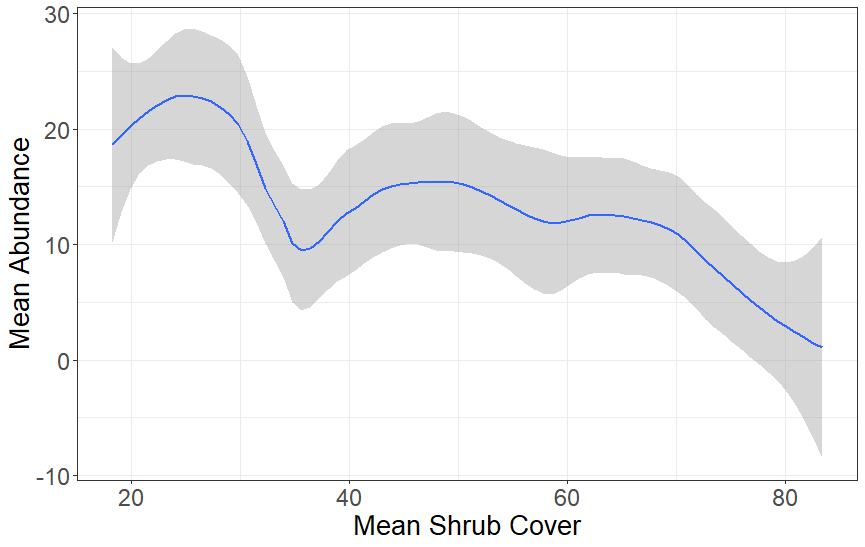

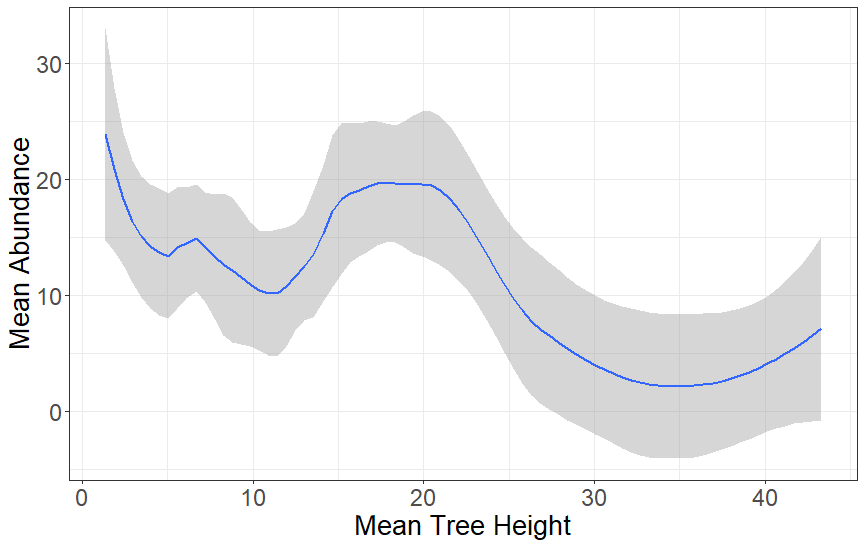

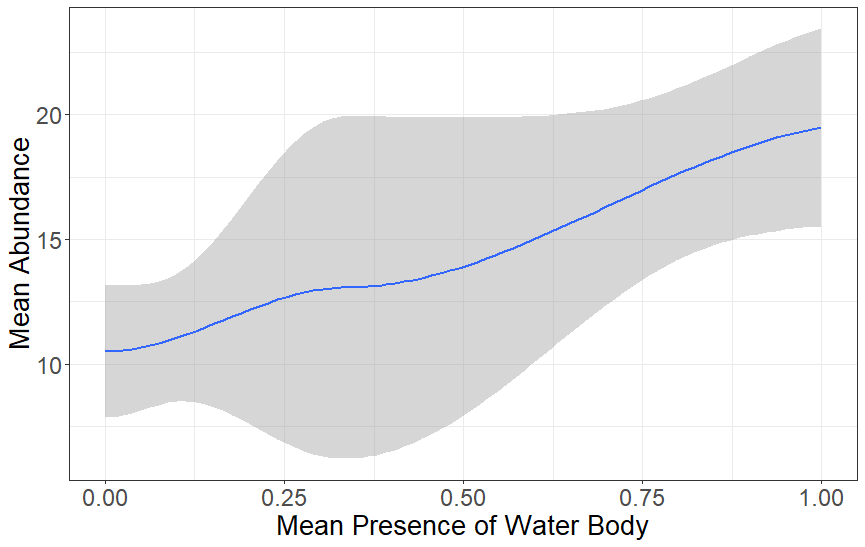

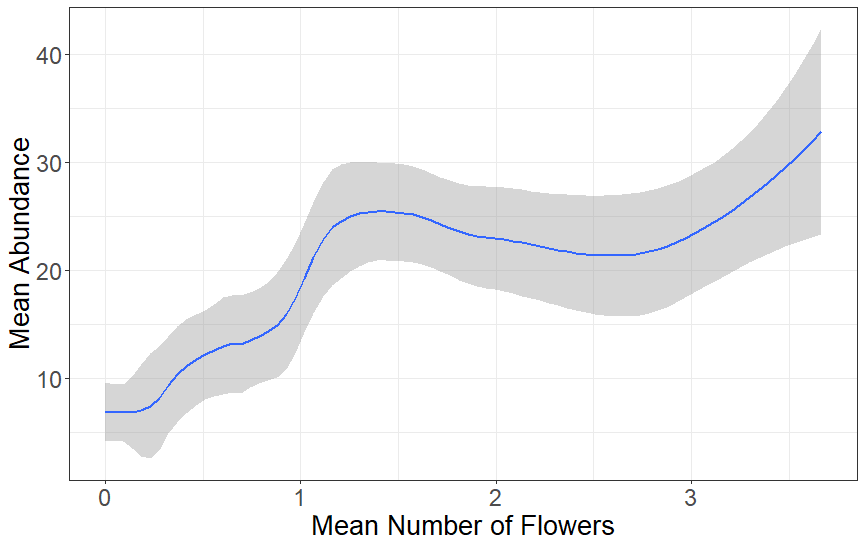


**Figure A5-3:** Line graphs of relationships between the Simpson Index and habitat traits.


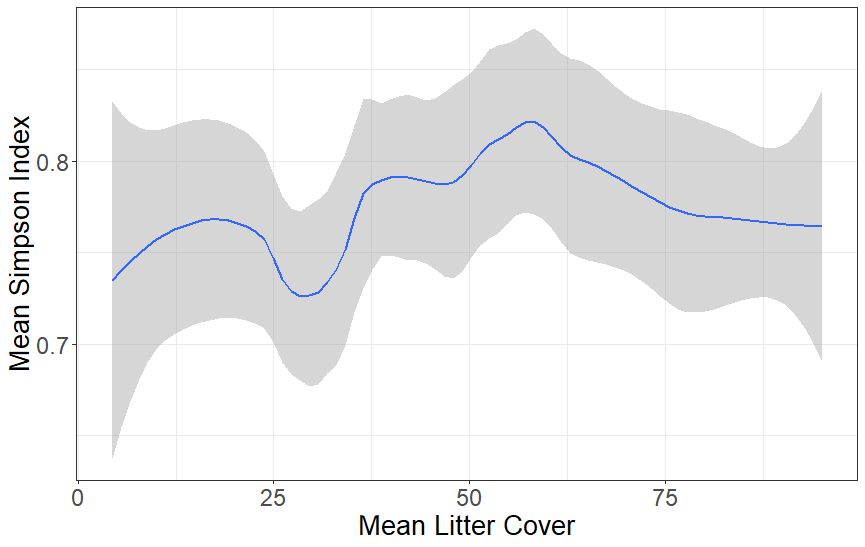

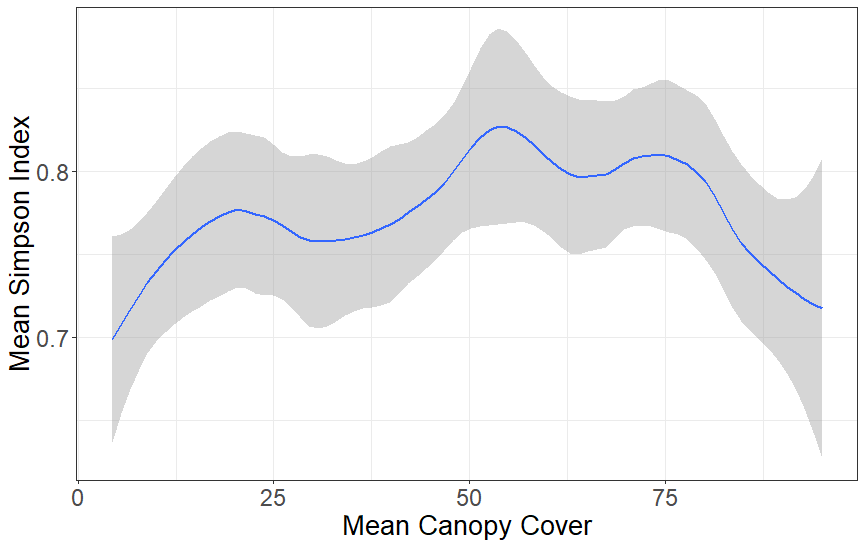

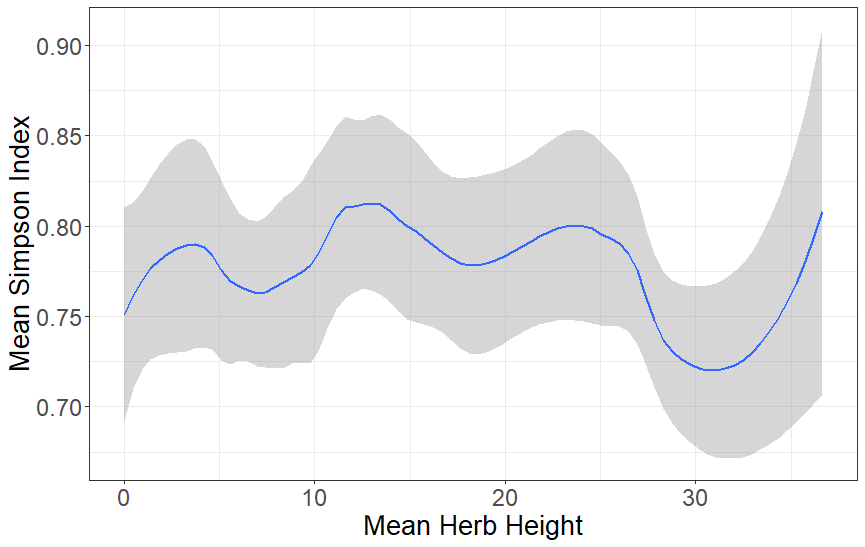

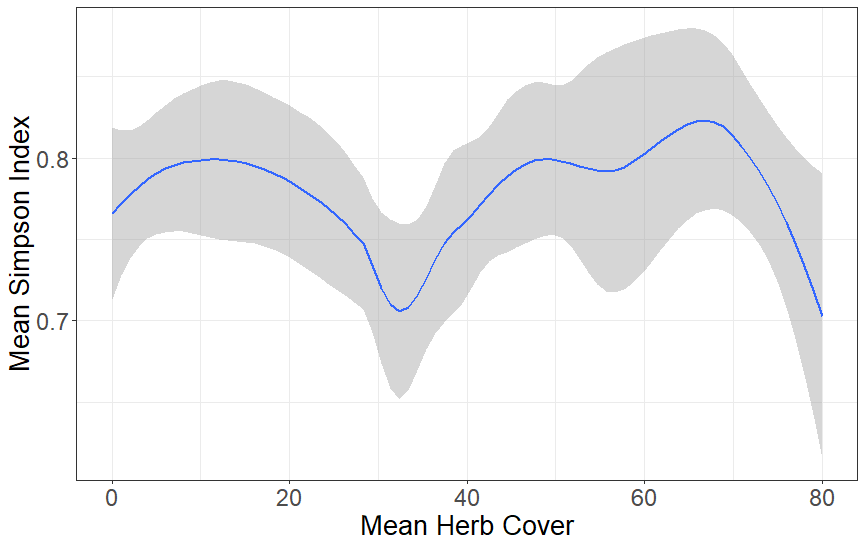

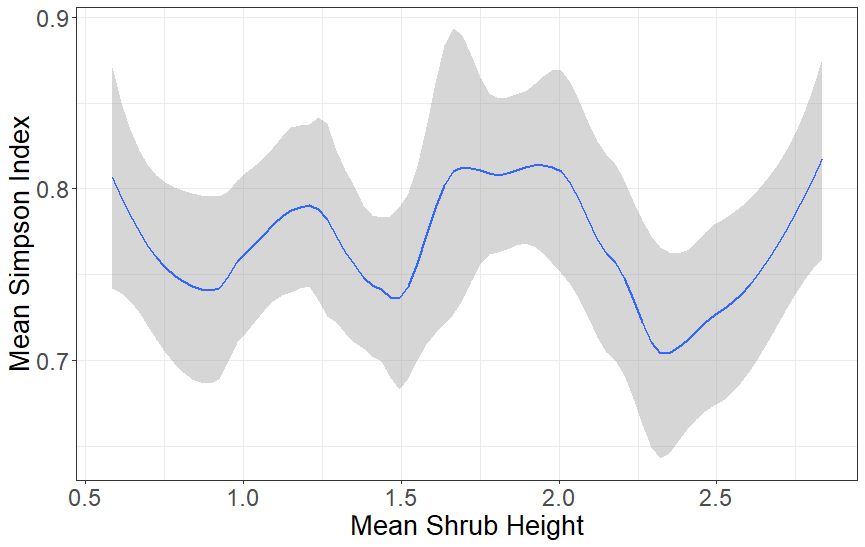

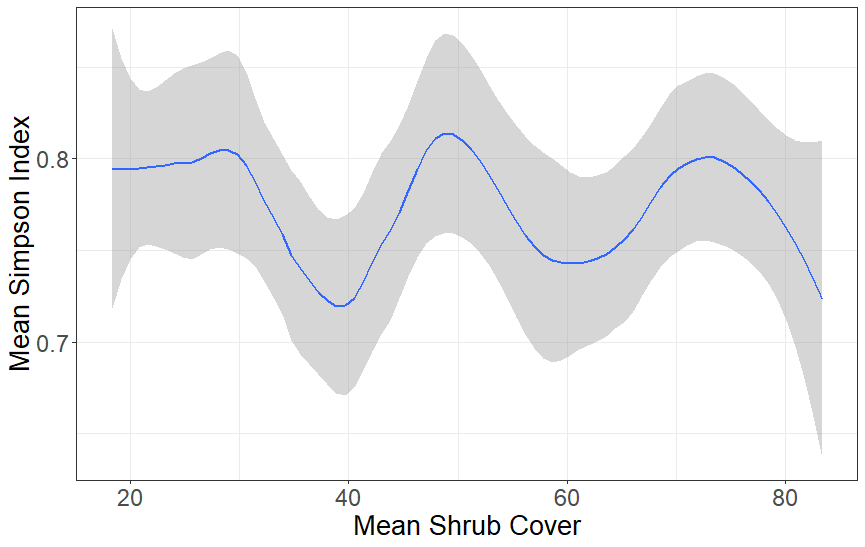

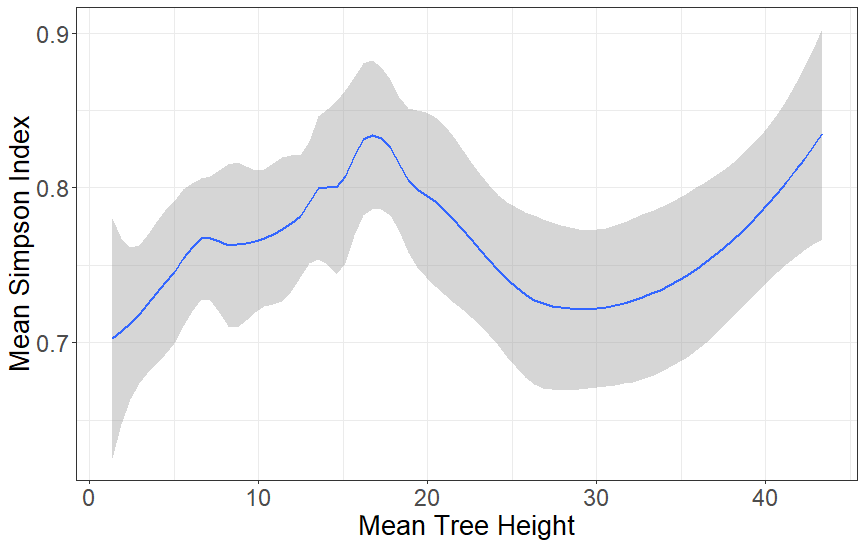

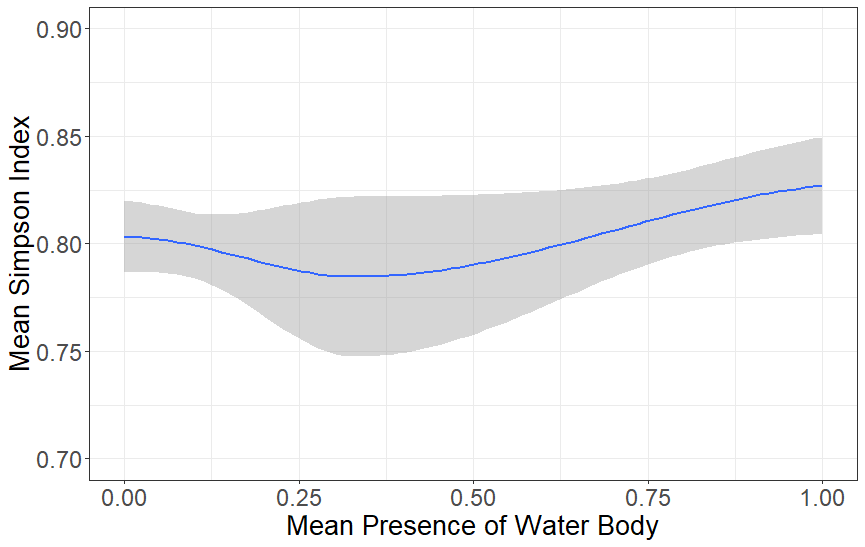

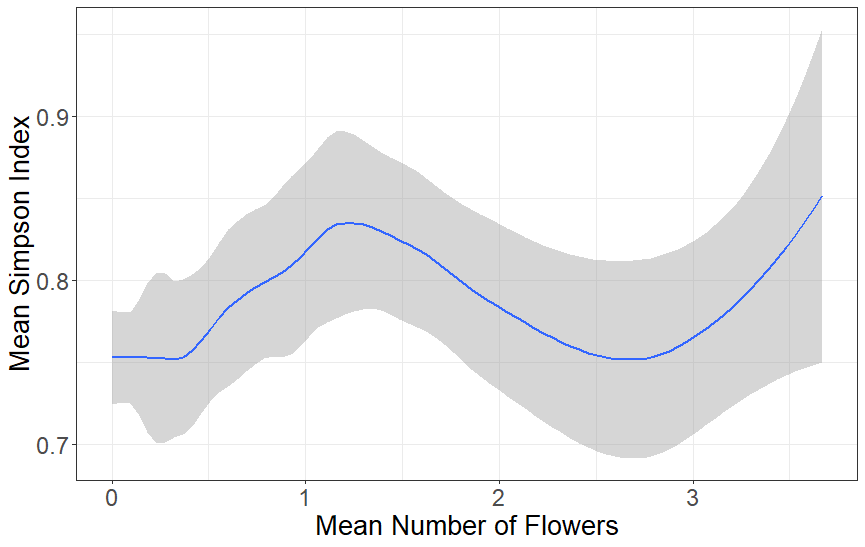


**Figure A5-4:** Line graphs of relationships between the Shannon index and habitat traits.


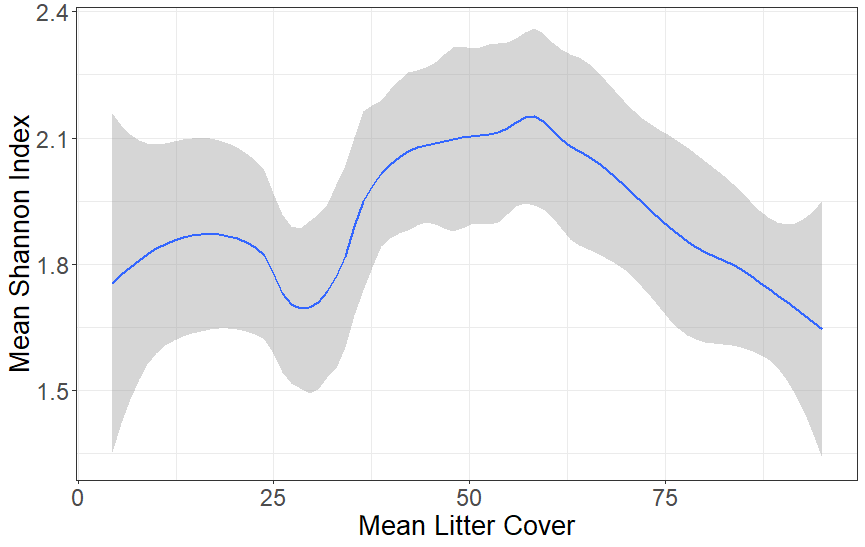

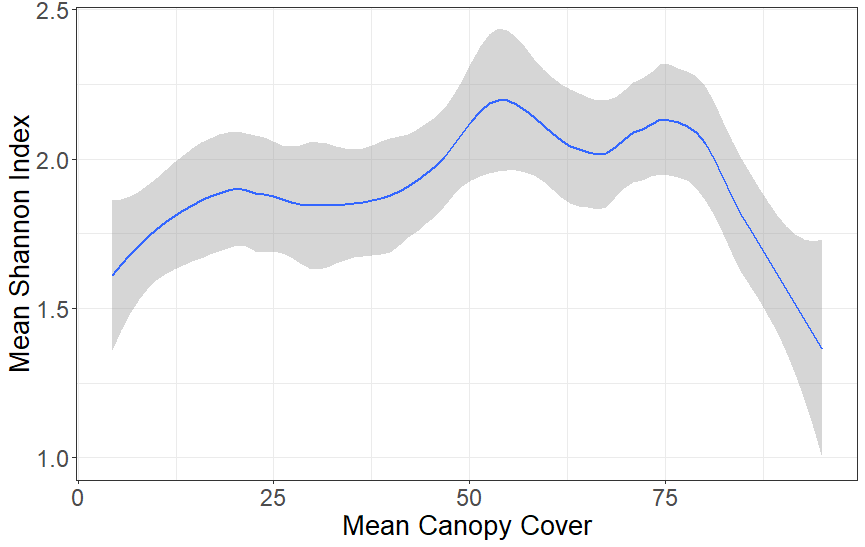


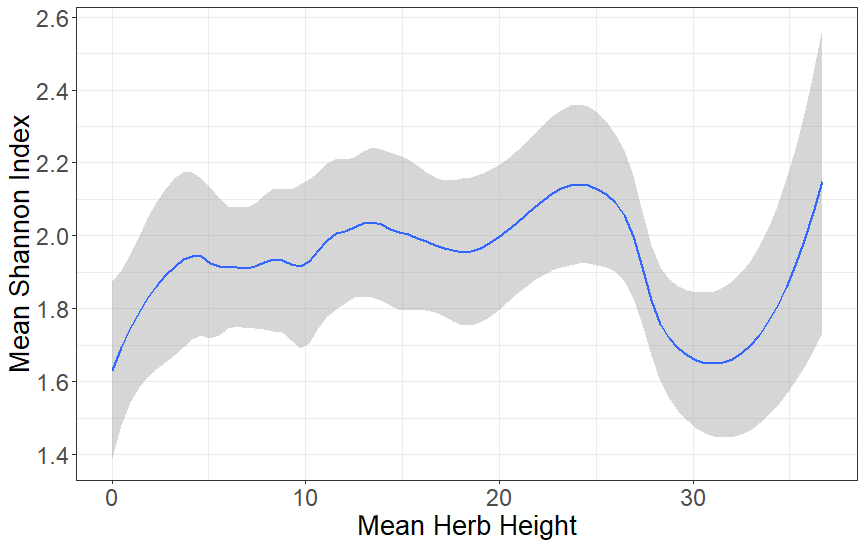

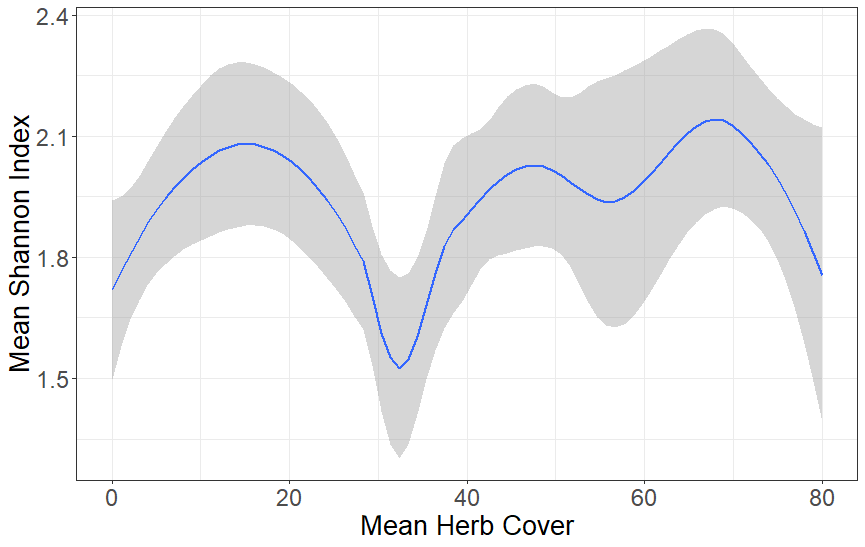

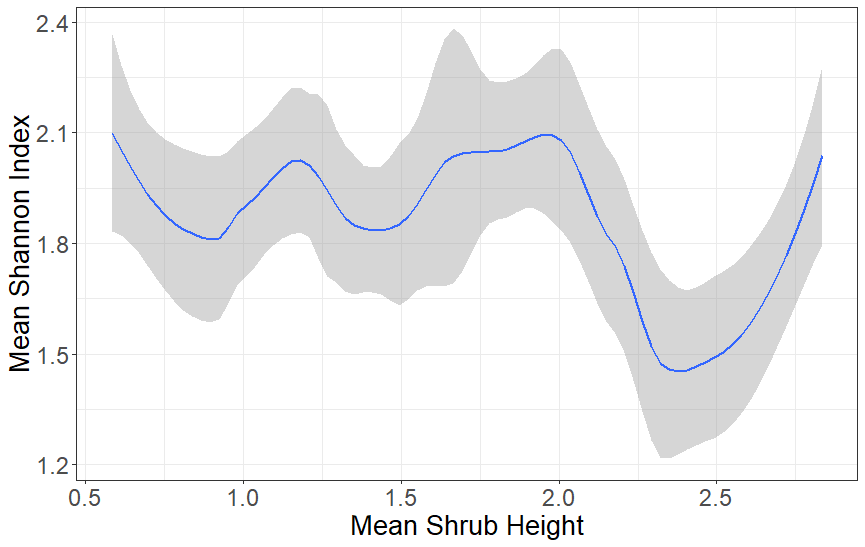

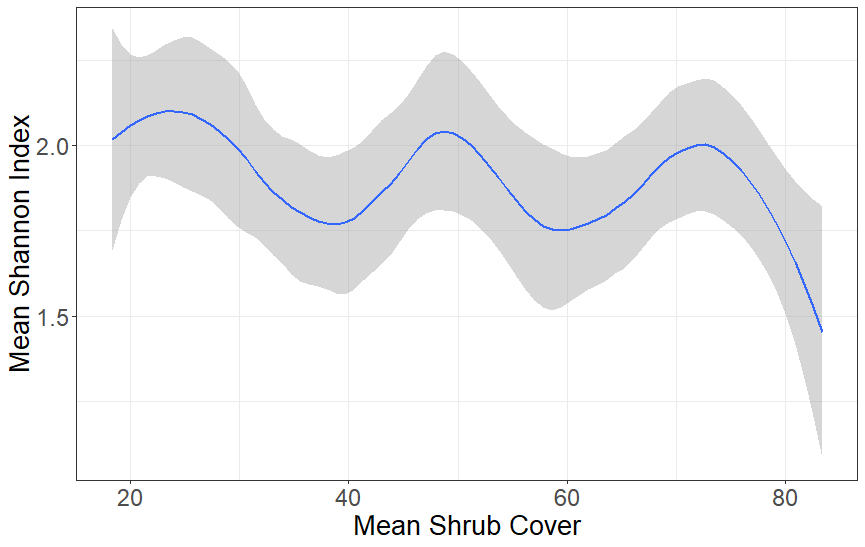

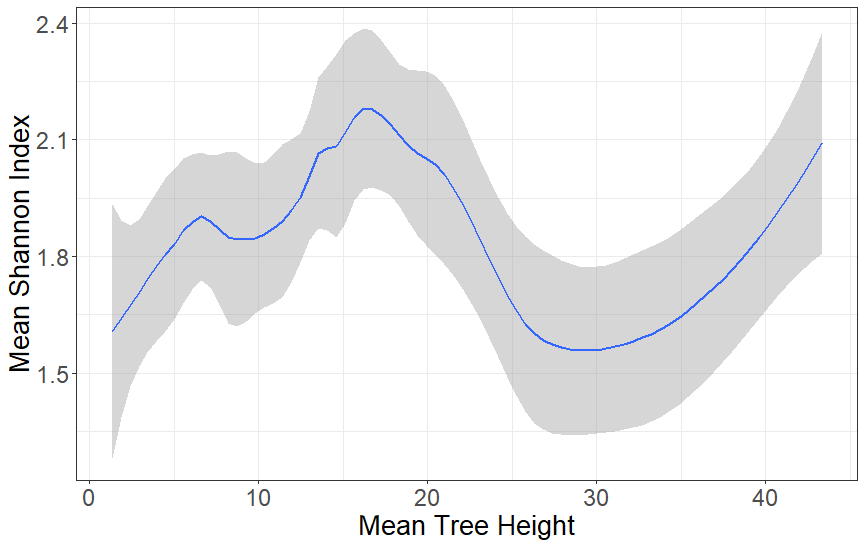

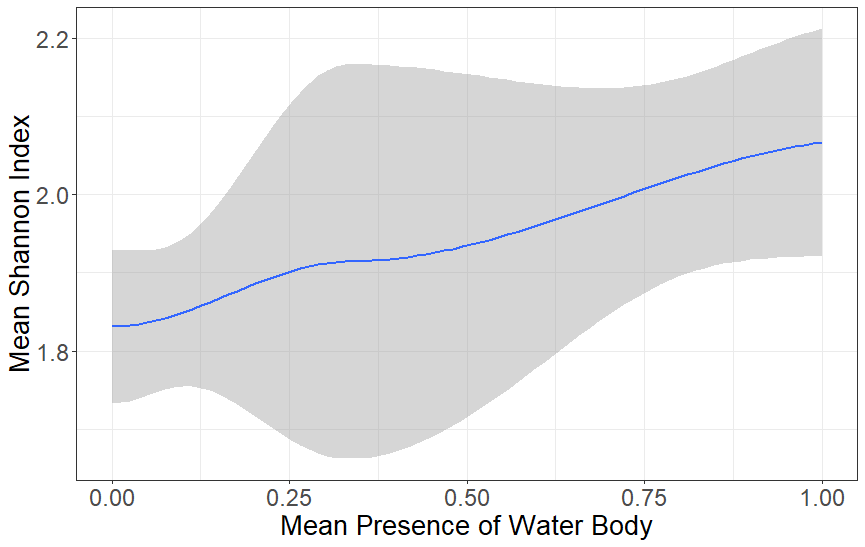

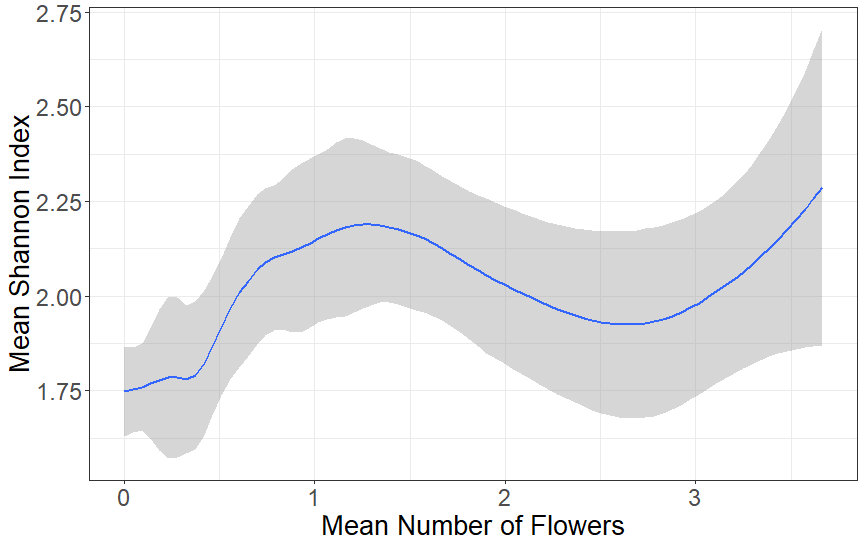


**Figure A5-5:** Line graphs of relationships between Evenness and habitat traits


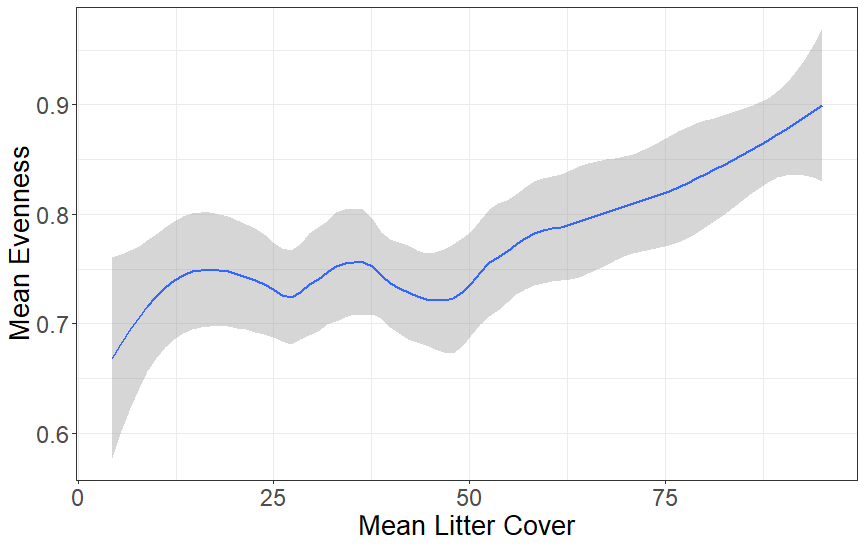

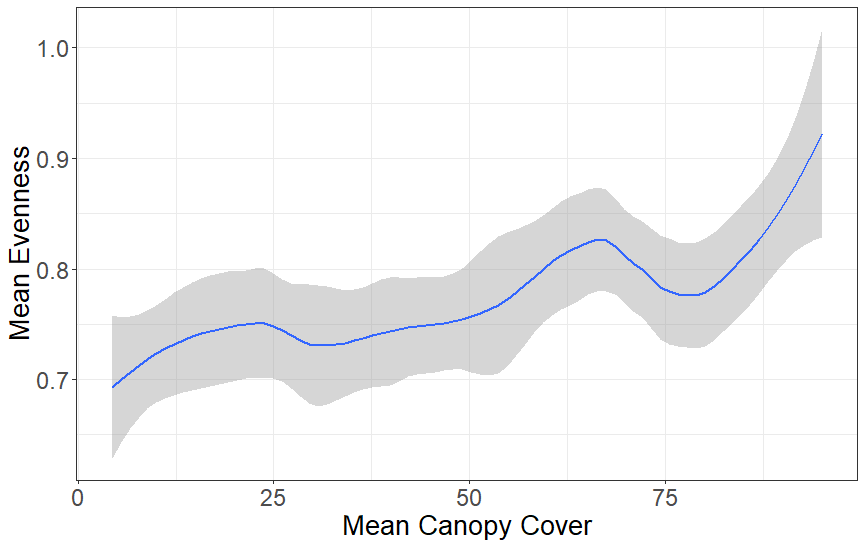

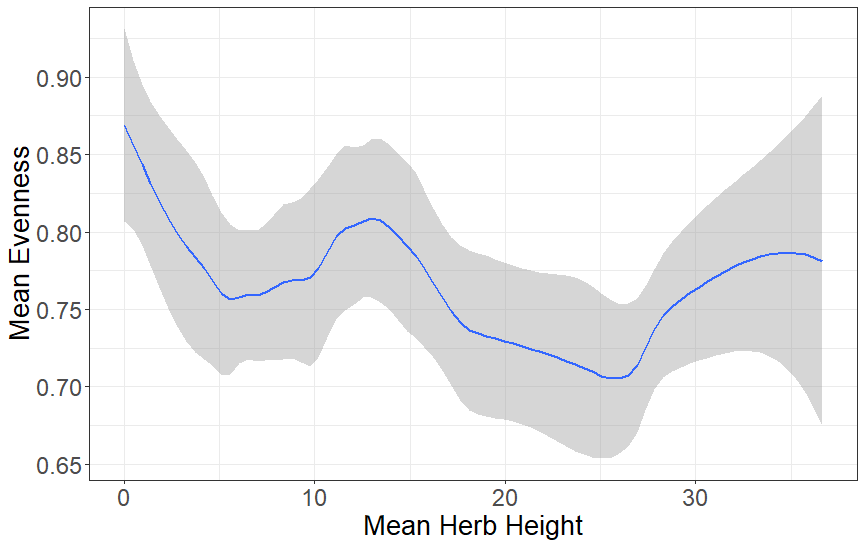

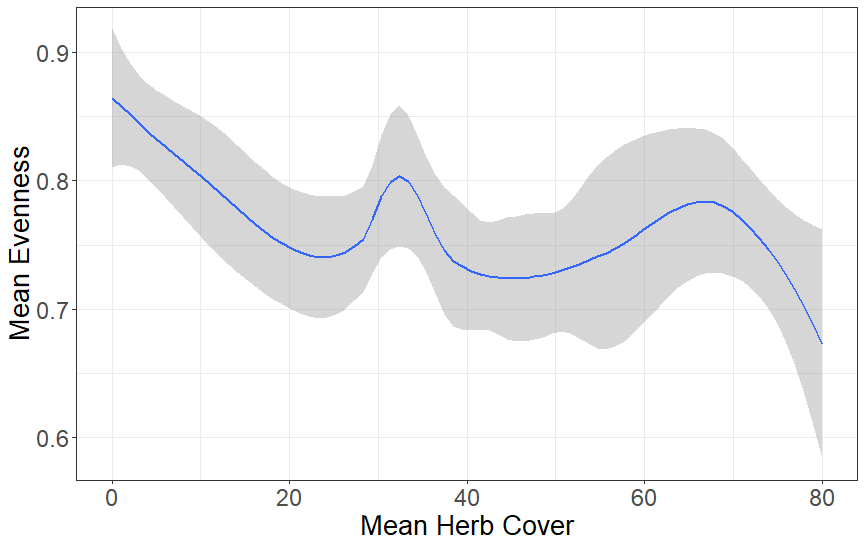

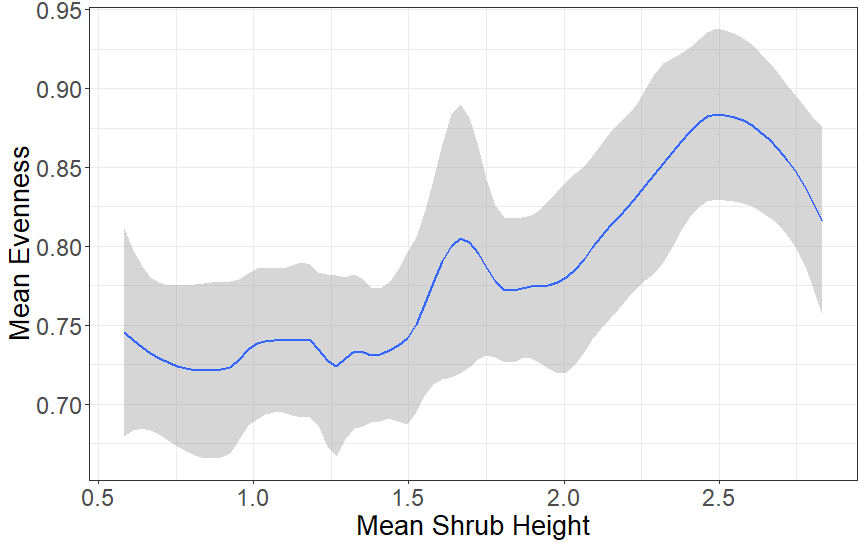

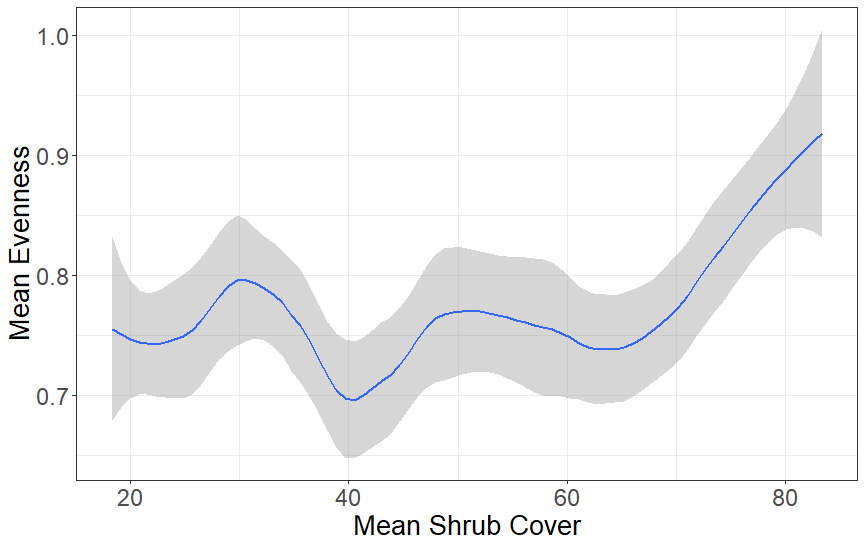

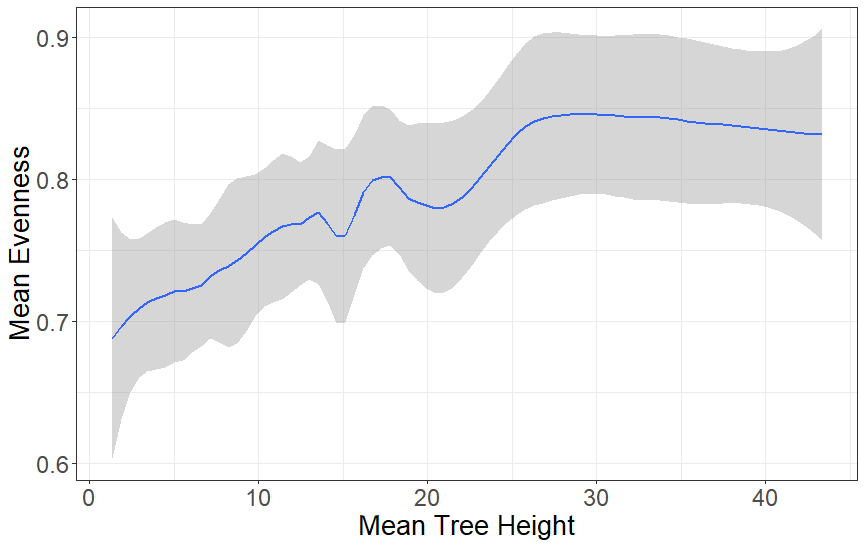

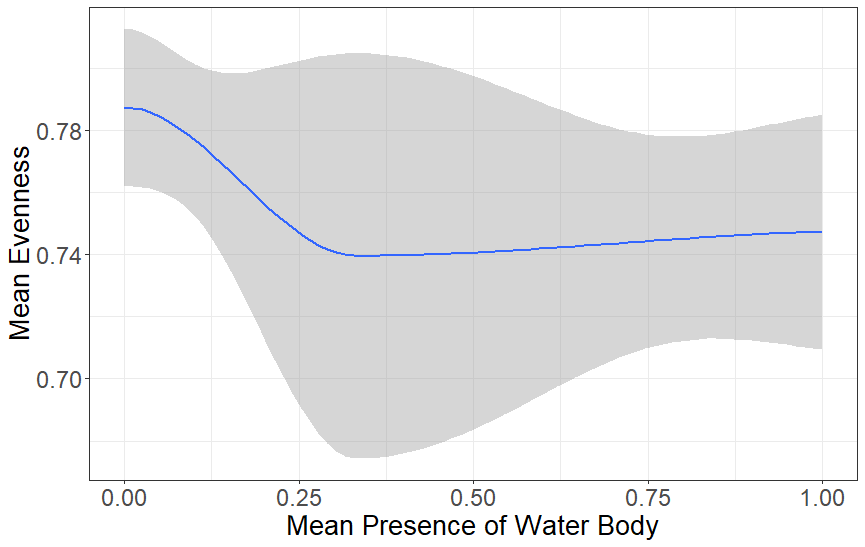

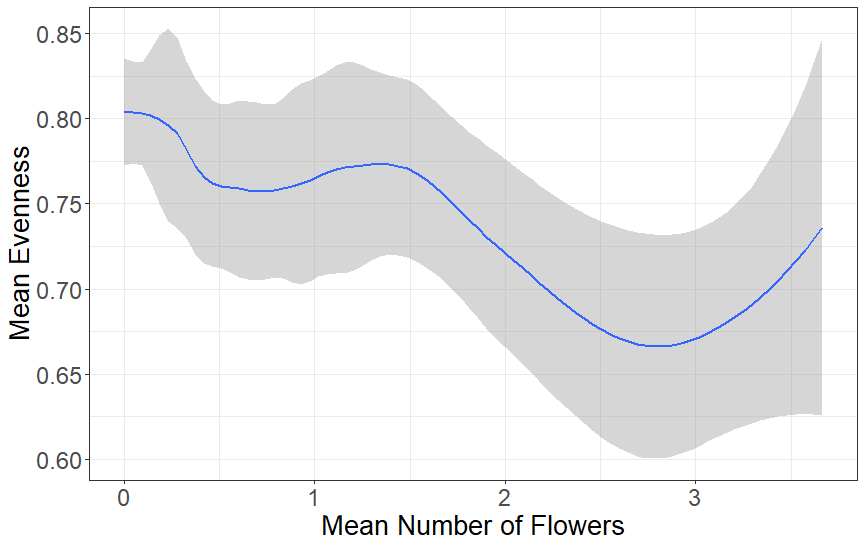

Supplement: Supplementary file 5 — Appendix S5: ece373242‐sup‐0005‐Appendix5.docx. [file ECE3-16-e73242-s004.docx]
